# A Novel Serum‐Based Diagnosis of Alzheimer's Disease Using an Advanced Phage‐Based Biochip

**DOI:** 10.1002/advs.202301650

**Published:** 2023-05-07

**Authors:** Maria Giovanna Rizzo, Laura Maria De Plano, Nicoletta Palermo, Domenico Franco, Marco Nicolò, Emanuele Luigi Sciuto, Giovanna Calabrese, Salvatore Oddo, Sabrina Conoci, Salvatore P. P. Guglielmino

**Affiliations:** ^1^ Department of Chemical Biological Pharmaceutical and Environmental Sciences University of Messina Viale F. Stagno d'Alcontres 31 Messina 98166 Italy; ^2^ Department of Chemistry G. Ciamician – Via F. Selmi 2 University of Bologna Bologna 40126 Italy; ^3^ LAB Sense Beyond Nano – DSFTM CNR Viale F. Stagno d'Alcontres 31 Messina 98166 Italy; ^4^ STMicroelectronics Stradale Primosole 50 Catania 95121 Italy

**Keywords:** A‐beta, biomarker, biosensor, neurodegeneration, phage display

## Abstract

55 million people worldwide suffer from Alzheimer's disease (AD). A definitive diagnosis of AD is made postmortem after a neuropathological examination of the brain. There is an urgent need for an innovative, noninvasive methodology that allows for an early and reliable diagnosis. Several engineered phages that recognized A*β*‐autoantibodies present in the sera of AD patients are previously identified. Here, novel phages are tested for their ability to accurately discriminate AD sera using immunophage‐polymerase chain reaction in a miniatured biochip. It is found that five of the six phages analyzed discriminate between healthy controls and AD patients. Further, by combining the response of two phages, non‐AD and severe AD cases are identified with 100% accuracy and mild‐to‐moderate cases with 90% accuracy. While the number of cases used here are relatively small and can be confirmed in larger cohorts, this first‐of‐a‐kind system represents an innovative methodology with the potential of having a major impact in the AD field: from a clinical perspective, it can aid physicians in making an accurate AD diagnosis; from a research perspective, it can be used as a surrogate for AD clinical trials.

## Graphical abstract

This paper describes the development of an innovative system for the noninvasive diagnosis of Alzheimer's disease (AD) using specifically engineered phages integrated into a miniaturized biochip. The system discriminates different stages of AD with an accuracy of >97%. This first‐of‐a‐kind system may aid physicians in making an accurate diagnosis and it may be used as a surrogate for clinical trials.

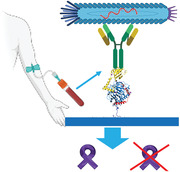

## 1 Introduction

Alzheimer's disease (AD) is the most common neurodegenerative disorder that affects more than 55 million people in the world.^[^ ^1^, ^2^ ^]^ Clinically, it manifests with memory and behavioral alterations that eventually impair patients’ daily life activities.^[^ ^3^ ^]^ Pathologically, AD is characterized by the accumulation of plaques and tangles. The former are mainly made of a small peptide called amyloid‐*β*. The latter are made of the hyperphosphorylated protein tau.^[^ ^3^ ^]^ The vast majority of AD cases are sporadic and while there are clear risk factors associated with the disease, the etiology remains unknown. In contrast, a small percentage of cases are due to dominant mutations in one of three genes: amyloid precursor protein, presenilin 1, and presenilin 2.^[^ ^3^ ^]^

While there are no effective treatments for AD,^[^ ^4^ ^]^ overwhelming evidence indicates that selective lifestyle changes (e.g., reducing exposure to known risk factors) can significantly decrease the probability of developing the disease or delay its onset.^[^ ^1^, ^2^ ^]^ However, AD must be diagnosed early for them to be effective. Indeed, it is well established that pathological changes in the brain precede, sometimes even by decades, the clinical manifestation of the disease.^[^ ^5^, ^6^ ^]^ Unfortunately, by the time a patient sees a specialist, the brain is already irreversibly compromised.^[^ ^6^, ^7^ ^]^ Despite this evidence, there is a lack of easy, inexpensive, and noninvasive practices that would allow for an early diagnosis, even at the primary physician's office. Indeed, today the goal standard to aid neurologists in making a proper diagnosis is the use of amyloid positron emission tomography (PET) imaging, which allows quantifying the amount of A*β* plaques in patients’ brains. However, this is a very expensive procedure and is not always accurate, given the lack of correlation between brain amyloidosis and the clinical manifestation of the disease.^[^ ^8^ ^]^ Along these lines, overwhelming evidence indicates that smaller A*β* aggregates (i.e., A*β* oligomers) are more toxic than A*β* plaques, which are the target of amyloid PET imaging.^[^ ^9^, ^10^ ^]^

Identifying peripheral biomarkers that allow for an early and correct diagnosis of AD is an active area of investigation. Much attention has been placed on the presence of A*β* autoantibodies in sera and cerebrospinal fluid of AD patients.^[^ ^11^, ^12^ ^]^ While early reports failed to highlight a consistent correlation between disease status and levels of circulating A*β* autoantibodies,^[^ ^12^ ^]^ more recently a positive association between the amount of A*β* autoantibodies in the sera of AD patients and the disease state has been observed.^[^ ^13^, ^14^ ^]^

Phage display is a widely used high‐throughput technique to identify short peptides that selectively recognize and bind to a target molecule (e.g., another protein or an antibody).^[^ ^15^ ^]^ These libraries have been often used for screening disease‐specific antigen mimics, including A*β* autoantibodies.^[^ ^16^, ^17^, ^18^ ^]^ In this work, we used six phages, each expressing a unique small peptide on its surface, which recognize conformation‐specific IgG1 A*β* autoantibodies, to determine if they could be used to discriminate between different stages of AD based on the amount of A*β* autoantibodies in the sera.

## 2 Results

**Figure 1:**
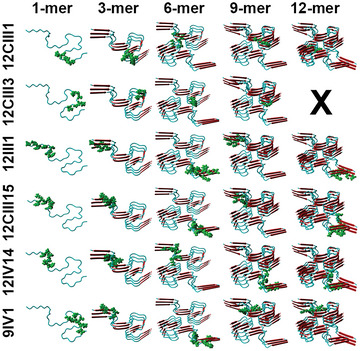
Predictive interaction between A*β* and the peptide exposed by the engineered phages. The picture shows the structure of different assembly states of A*β* obtained by remodeling YASARA software. Each structure is shown in cartoon style with *β*‐sheets (red) and *α*‐helix (light blue). The homologous amino acids to phage‐peptides are shown in green.

We screened an M13 pVIII phage library using a dual biopanning approach and identified and tested six phages that recognize auto‐antibodies in sera of AD patients.^[^ ^16^ ^]^ Each of these phages has either a 9‐ or a 12‐amino acid peptide, with a unique sequence (**Table** **1**), expressed as a fusion protein with the phage pVIII protein and thus exposed outside the capsid. To assess whether these phages have the potential to directly bind different assembly states of A*β*, we used a web‐based analytical tool, PepSurf, which allows us to map the interaction between a peptide and a known 3D structure.^[^ ^19^ ^]^ Specifically, we used the sequence of the peptide exposed by each phage clone against a known 3D structure of A*β* (1‐mer, 3‐mer, 6‐mer, 9‐mer, and 12‐mer) that we obtained from ref. [20]. When several peptides are aligned, the server uses a clustering algorithm to detect one or more patches of residues on the surface of the surveyed protein.^[^ ^19^ ^]^ We found that the 3D structures of the six peptides are similar to the structures of the low molecular weight of A*β* oligomers (**Figure** **1**). This model indicates that the autoantibodies against A*β* should also recognize the engineered peptides. The only exception was for 12CIII3, whose peptide does not align to A*β* 12‐mer (Figure 1). Except for 9IV1, all clones had excellent alignment scores, indicating a strong alignment between A*β* and the peptide analyzed (Table 1). To determine if a phage had more affinity for a specific assembly state of A*β*, we compared the individual alignment scores of each clone with the different forms of A*β*. Our analysis indicated that five of the six phages studied had their weakest affinity with monomeric A*β* (Table 1). This is consistent with the selection method we used to identify these phages. Indeed, we used a double‐binding selection to screen our phage library against the F1 capsular antigen of Yersinia pestis (YFP19) and anti‐IgGs of AD patients, given the structural similarities between YFP19 and A*β* oligomers. In other words, it is likely the phages within our library that bind to monomeric A*β* were excluded during the biopanning process.^[^ ^16^ ^]^ Notably, while phage 12CIII3 recognized the mid‐to‐C terminal region of A*β*, all other phages mainly recognized the N‐terminal region of A*β* (Table S1, Supporting Information).

**Table 1:** The table shows the p. alignment score obtained by Pepsurf analysis between different conformation states of A*β* structures and the phage clones. The score values represent the affinity of phage peptides, in terms of their physicochemical properties and spatial organization on the different states of A*β*. The sequence of the peptide exposed by each phage clone is shown under the name of the phages

	p. alignment score					
Phage A*β*	**12CIII1** *GGGCIEGPCLEG*	**12CIII3** *WVGCHGEWCGVW*	**12III1** *RWPPHFEWHFDD*	**12III15** *WEYDRYRGWHIG*	**12IV14** *GGHWEWHADYNL*	**9IV1** *YNTIPSRRV*
**1‐mer**	12.6804	9.85962	13.4039	10.4418	10.3317	8.76848
**3‐mer**	16.642	12.4062	14.1152	14.1107	14.8228	8.13185
**6‐mer**	17.6894	12.4062	14.6903	15.3003	17.6901	8.26852
**9‐mer**	18.8821	12.4062	16.2536	16.158	17.6901	8.61988
**12‐mer**	18.8821	//	15.688	16.6289	17.6901	9.06916

**Figure 2:**
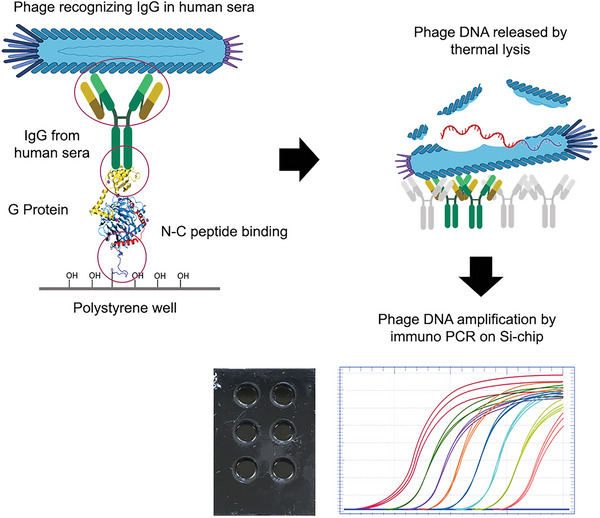
Phage‐mediated Immuno‐PCR. Diagram of the strategy used to quantify the amount of phage bound to human sera. The picture shows the phage sandwich recognizing IgG in human sera followed by the release of phage DNA by thermal lysis and the amplification of phage DNA by immuno‐PCR on a silicon chip. The real‐time PCR curves are shown as a representative example and do not indicate the result of any specific phage.

**Figure 3:**
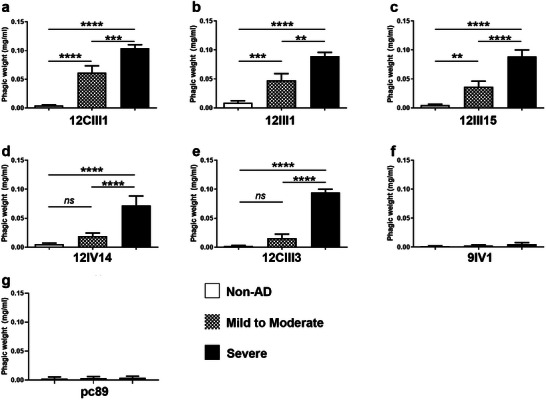
Most phages significantly discriminated among severe AD, mild‐to‐moderate AD, and non‐AD sera. a–f) The graphs report the quantitative analysis of the phagic weight across the different groups analyzed in this study. The phagic weight, which reflects the amount of conformational specific A*β* autoantibodies present in sera, was significantly different among the three groups, except phage 9IV1. g) pc89 was used as a negative control as it did not express any peptide on its surface. Consistently, pc89 did not recognize any autoantibody in the sera of any of the groups. Non‐AD *n* = 18; mild‐to‐moderate *n* = 11; severe AD *n* = 8. Data were analyzed by one‐way ANOVA followed by Bonferroni correction. ** indicates *p* < 0.01; *** indicates *p* < 0.001; **** indicates *p* < 0.0001; ns = nonsignificant.

To determine whether these six phages could discriminate between various stages of AD, we analyzed the sera of 18 AD patients (9 mild‐to‐moderate and 9 severe) and 18 healthy individuals (herein referred to as non‐AD). For the following analyses, we assigned a non‐AD status to individuals with a Mini‐Mental State Examination (MMSE) between 25 and 30; a mild‐to‐moderate status to individuals with an MMSE between 12 and 24; a severe status to individuals with MMSE <12 (Table S2, Supporting Information). We used a molecular sandwich made of sera IgG‐phages to quantify the amount of A*β* autoantibodies present in our sera. Specifically, we functionalized a 96‐well plate with protein G, after which we added the sera and completed the sandwich by adding the phages. To quantify the number of phages bound, which indirectly reflects the amount of A*β* autoantibodies present in sera, we lysed the phages and quantified their DNA through real‐time polymerase chain reaction (PCR) in a single‐step process performed on a silicon chip (**Figure** **2**). Given that protein G only recognizes IgG1, we can conclude that our phages only bind to this class of immunoglobulins. We found that for 12CIII1, 12III1, 12III15, 12IV14, and 12CIII3 the phagic weight was significantly different as indicated by one‐way analysis of variance (ANOVA, *p* < 0.0001 for all five phages). Post hoc analyses with Bonferroni's correction showed that 12CIII1, 12III1, and 12III15 discriminated among the three groups (i.e., all three groups were statistically significant from each other; *p* < 0.001 for all comparisons). However, Bonferroni's correction indicated that 12IV14 and 12CIII3 significantly discriminated between non‐AD and severe and between mild‐to‐moderate and severe (*p* < 0.0001 for all comparisons) but not between non‐AD and mild‐to‐moderate. Finally, 9IV1 and the wildtype phage, pc89, did not discriminate among the three groups (one‐way ANOVA *p* = 0.14 and 0.52, respectively; **Figure** **3**). Further, we tested the binding affinity of a representative phage (12III1) to the IgG A*β* autoantibodies. To this end, we incubated sera from severe‐AD with 12III1 in the presence or absence of 6 m urea and quantified the amount of phage through enzyme‐linked immunosorbent assay (ELISA) test. We found that incubation with urea did not significantly dissociate 12III1 from the IgG A*β* autoantibodies of the sera tested highlighting strong avidity of phage toward IgGs (Figure S1, Supporting Information, *p* > 0.05).

**Figure 4:**
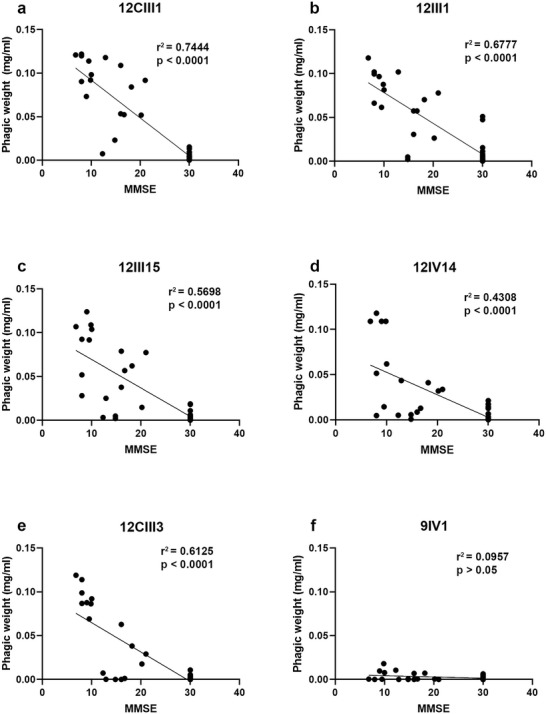
Negative correlation between phagic weight and MMSE scores. a–e) Pearson's correlation analyses indicated identified a negative correlation between the MMSE scores and 12CIII1, 12III1, 12III15, 12IV14, and 12CIII3. A lack of statistically significant correlation was evident for 9IV1.

Next, we performed a Pearson correlation to determine whether the phagic weight of each phage correlated with the MMSE. Consistent with the results obtained so far, we found a significant correlation with phages 12CIII1, 12III1, 12II15, 12IV14, and 12CIII3 (*r* ^2^ = 0.74, 0.68, 0.57. 0.43, 0.61, respectively; *p* < 0.0001 for all five phages). In contrast, a significant correlation was not evident for 9IV1 (**Figure** **4**).

**Figure 5:**
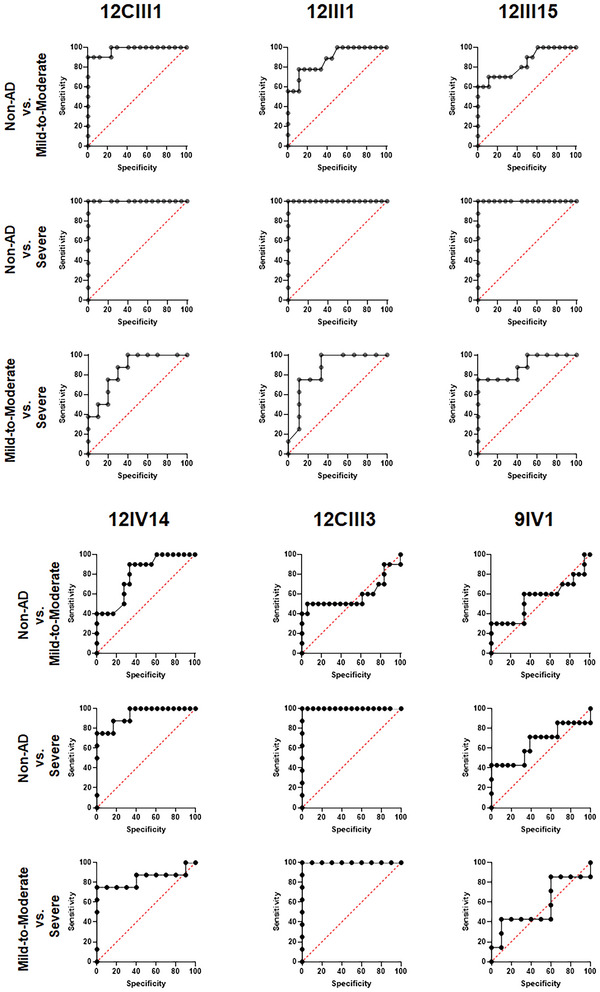
ROC analysis. The graphs report the ROC analyses for the six phages tested in this study. For each analysis, both the area under the curve and the *p* value are reported in the Results section.

Our results so far indicate that of the six phages analyzed, five detected sera A*β* autoantibody and showed a significantly greater response in severe AD, compared to the mild‐to‐moderate AD group, and the non‐AD group. Additionally, when tested individually, these five phages showed a significant correlation between their response and the MMSE scores. To assess the specificity and selectivity of the phage responses, we first assessed the area under the curve (AUC) for receiver operating characteristic (ROC) analysis (**Figure** **5**). The AUCs for 12CIII1 were 0.977 (*p* < 0.0001), 1.000 (*p* < 0.0001), and 0.850 (*p* = 0.013) when comparing non‐AD to mild‐to‐moderate, non‐AD to severe AD, and mild‐to‐moderate to severe AD, respectively. The AUCs for 12III1 were 0.883 (*p* = 0.0014), 1.000 (*p* < 0.0001), and 0.854 (*p* = 0.014) when comparing non‐AD to mild‐to‐moderate, non‐AD to severe AD, and mild‐to‐moderate to severe AD, respectively. The AUCs for 12CIII15 were 0.842 (*p* = 0.0032), 1.000 (*p* < 0.0001), and 0.887 (*p* = 0.006) when comparing non‐AD to mild‐to‐moderate, non‐AD to severe AD, and mild‐to‐moderate to severe AD, respectively. The AUCs for 12IV14 were 0.797 (*p* = 0.0103), 0.9375 (*p* = 0.0005), and 0.8375 (*p* = 0.016) when comparing non‐AD to mild‐to‐moderate, non‐AD to severe AD, and mild‐to‐moderate to severe AD, respectively. The AUCs for 12CIII3 were 0.5917 (*p* = 0.429), 1.000 (*p* < 0.0001), and 1.000 (*p* < 0.0001) when comparing non‐AD to mild‐to‐moderate, non‐AD to severe AD, and mild‐to‐moderate to severe AD, respectively. The AUCs for 9IV1 were 0.5583 (*p* = 0.615), 0.6587 (*p* < 0.226), and 0.571 (*p* = 0.626) when comparing non‐AD to mild‐to‐moderate, non‐AD to severe AD, and mild‐to‐moderate to severe AD, respectively. To further analyze the ability of our phages to discriminate among these three groups of individuals, we performed a discriminant analysis by first analyzing each phage individually. We found that 12CIII1 and 12CIII3 were the two top‐performing phages as they correctly discriminated 83.33% and 77.78% of the tested sera. Specifically, 12CIII1 recognized the non‐AD cases with 100% confidence, the mild‐to‐moderate cases with 50% confidence, and the severe cases with 87.5% confidence (**Table** **2** and Figure S2 and Table S3, Supporting Information). In contrast, 12CIII3 identified correctly 100% of the severe AD cases, 94.44% of the non‐AD cases, and only 30% of the mild‐to‐moderate (Table 2 and Figure S2 and Table S3, Supporting Information). This pattern was evident for all phages analyzed. In other words, while all phages (except for 9IV1) discriminated non‐AD cases or severe AD cases with a high degree of confidence, they were less successful at classifying cases within the mild‐to‐moderate AD group (Table 2 and Figure S2 and Tables S4–S8, Supporting Information). Consistent with the data reported so far, 9IV1 was the less performing phage as it was successful at identifying the proper group only 88.89%, 20%, and 37.5% of the cases for non‐AD, mild‐to‐moderate, and severe, respectively (Table 2 and Figure S2 and Table S8, Supporting Information).

**Table 2:** Confusion matrix for 36 sera (18 non‐AD, 10 mild‐to‐moderate AD, and 8 severe AD) for each phage clone

12CIII1						12III1					
from ∖ to	Non‐AD	Mild‐to‐moderate AD	Severe AD	Total	% Correct	from ∖ to	Non‐AD	Mild‐to‐moderate AD	Severe AD	Total	% Correct
Non‐AD	18	0	0	18	100.00%	Non‐AD	16	2	0	18	88.89%
Mild‐to‐moderate AD	1	5	4	10	50.00%	Mild‐to‐moderate AD	4	3	3	10	30.00%
Severe AD	0	1	7	8	87.50%	Severe AD	0	2	6	8	75.00%
Total	19	6	11	36	83.33%	Total	20	7	9	36	69.44%

12III15						12IV14					
from ∖ to	Non‐AD	Mild‐to‐moderate AD	Severe AD	Total	% Correct	from ∖ to	Non‐AD	Mild‐to‐moderate AD	Severe AD	Total	% Correct
Non‐AD	16	2	0	18	88.89%	Non‐AD	16	2	0	18	88.89%
Mild‐to‐moderate AD	4	4	2	10	40.00%	Mild‐to‐moderate AD	6	4	0	10	40.00%
Severe AD	0	2	6	8	75.00%	Severe AD	2	0	6	8	75.00%
Total	20	8	8	36	72.22%	Total	24	6	6	36	72.22%

12CIII3						9IV1					
from ∖ to	Non‐AD	Mild‐to‐moderate AD	Severe AD	Total	% Correct	from ∖ to	Non‐AD	Mild‐to‐moderate AD	Severe AD	Total	% Correct
Non‐AD	17	1	0	18	94.44%	Non‐AD	16	2	0	18	88.89%
Mild‐to‐moderate AD	6	3	1	10	30.00%	Mild‐to‐moderate AD	7	2	1	10	20.00%
Severe AD	0	0	8	8	100.00%	Severe AD	5	0	3	8	37.50%
Total	23	4	9	36	77.78%	Total	23	4	9	36	58.33%

To determine if the combination of two or more phages together would improve the specificity and sensitivity of our system, we performed a discriminant analysis to combine the individual response of two or more phages (Figure S3 and Tables S9–S18, Supporting Information). We found that the highest overall accuracy was reached when we combined phage 12CIII1 and 12CIII3 (Figure S3d, Supporting Information, **Table** **3**a, and Table S12, Supporting Information). Indeed, these two phages correctly classified 100% of non‐AD cases, 100% of severe AD cases, and 90% of mild‐to‐moderate cases (Table 3a and Table S12, Supporting Information). That is, out of the 36 sera analyzed, these two phages correctly classified 35 of them. The only misclassification was linked to a mild‐to‐moderate case with an MMSE of 12.3, which the system classified as a non‐AD case. Notably, adding a third clone to the pair 12CIII1/12CIII3 did not further improve the accuracy of the system (Figure S4, Supporting Information, Table 3b, and Tables S19–S21, Supporting Information). When we combined all clones, we obtained 100% accuracy for all three sera groups (Figure S4d and Table S22, Supporting Information). However, it should be noted that the only serum wrongly classified by the pair 12CIII1 and 12CIII3 (serum #25) was properly classified by combining all sera together with only a 50.3% confidence (Table S22, Supporting Information). Despite the relatively small number of cases, these data indicate that the combination of two close phages, 12CIII1 and 12CIII3, classify with higher accuracy different stages of AD based on the presence of A*β* autoantibodies in the sera. Our results are exciting but will have to be confirmed using a larger cohort of patients.

**Table 3:** Confusion matrix for 36 sera (18 non‐AD, 10 mild‐to‐moderate AD, and 8 severe AD)

a) Two phage clones											
12CIII1 + 12III1						12CIII1 + 12III15					
from ∖ to	Non‐AD	Mild‐to‐moderate AD	Severe AD	Total	% Correct	from ∖ to	Non‐AD	Mild‐to‐moderate AD	Severe AD	Total	% Correct
Non‐AD	18	0	0	18	100.00%	Non‐AD	18	0	0	18	100.00%
Mild‐to‐moderate AD	1	6	3	10	60.00%	Mild‐to‐moderate AD	1	6	3	10	60.00%
Severe AD	0	0	8	8	100.00%	Severe AD	0	1	7	8	87.50%
Total	19	6	11	36	88.89%	Total	19	7	10	36	86.11%

12CIII1 + 12IV14						12CIII1 + 12CIII3					
from ∖ to	Non‐AD	Mild‐to‐moderate AD	Severe AD	Total	% Correct	from ∖ to	Non‐AD	Mild‐to‐moderate AD	Severe AD	Total	% Correct
Non‐AD	18	0	0	18	100.00%	Non‐AD	18	0	0	18	100.00%
Mild‐to‐moderate AD	1	8	1	10	80.00%	Mild‐to‐moderate AD	1	9	0	10	90.00%
Severe AD	0	1	7	8	87.50%	Severe AD	0	0	8	8	100.00%
Total	19	9	8	36	91.67%	Total	19	9	8	36	97.22%

12III1 + 12III15						12III1 + 12IV14					
from ∖ to	Non‐AD	Mild‐to‐moderate AD	Severe AD	Total	% Correct	from ∖ to	Non‐AD	Mild‐to‐moderate AD	Severe AD	Total	% Correct
Non‐AD	15	3	0	18	88.89%	Non‐AD	16	2	0	18	88.89%
Mild‐to‐moderate AD	3	5	2	10	80.00%	Mild‐to‐moderate AD	3	7	0	10	70.00%
Severe AD	0	1	7	8	100.00%	Severe AD	0	1	7	8	87.50%
Total	18	9	9	36	88.89%	Total	19	10	7	36	83.33%

12III1 + 12CIII3						12III15 + 12IV14					
from ∖ to	Non‐AD	Mild‐to‐moderate AD	Severe AD	Total	% Correct	from ∖ to	Non‐AD	Mild‐to‐moderate AD	Severe AD	Total	% Correct
Non‐AD	16	2	0	18	88.89%	Non‐AD	18	0	0	18	100.00%
Mild‐to‐moderate AD	2	8	0	10	80.00%	Mild‐to‐moderate AD	3	7	0	10	70.00%
Severe AD	0	0	8	8	100.00%	Severe AD	0	3	5	8	62.50%
Total	18	10	8	36	88.89%	Total	21	10	5	36	83.33%

12III15 + 12CIII3						12CIII1 + 12III15					
from ∖ to	Non‐AD	Mild‐to‐moderate AD	Severe AD	Total	% Correct	from ∖ to	Non‐AD	Mild‐to‐moderate AD	Severe AD	Total	% Correct
Non‐AD	17	1	0	18	94.44%	Non‐AD	17	1	0	18	94.44%
Mild‐to‐moderate AD	3	7	0	10	70.00%	Mild‐to‐moderate AD	5	5	0	10	50.00%
Severe AD	0	0	8	8	100.00%	Severe AD	0	0	8	8	100.00%
Total	20	8	8	36	88.89%	Total	22	6	8	36	83.33%

b) Three phage clones											
12CIII1/12III1 + 12III1						12CIII1/12III1 + 12III15					
from ∖ to	Non‐AD	Mild‐to‐moderate AD	Severe AD	Total	% Correct	from ∖ to	Non‐AD	Mild‐to‐moderate AD	Severe AD	Total	% Correct
Non‐AD	18	0	0	18	100.00%	Non‐AD	18	0	0	18	100.00%
Mild‐to‐moderate AD	1	9	0	10	90.00%	Mild‐to‐moderate AD	1	9	0	10	90.00%
Severe AD	0	0	8	8	100.00%	Severe AD	0	0	8	8	100.00%
Total	19	9	8	36	97.22%	Total	19	9	8	36	97.22%

12CIII1/12III1 + 12IV14											
from ∖ to	Non‐AD	Mild‐to‐moderate AD	Severe AD	Total	% Correct						
Non‐AD	18	0	0	18	100.00%						
Mild‐to‐moderate AD	1	9	0	10	90.00%						
Severe AD	0	0	8	8	100.00%						
Total	19	9	8	36	97.22%						

## 3 Discussion

Standard phage libraries are made of bacteriophages that express random peptides as fusion proteins with one of their capsid proteins.^[^ ^21^ ^]^ One of the major advantages of these libraries is the extremely large number of peptides (often > 10^12^) present in the library.^[^ ^22^ ^]^ This allows for the screening of phage libraries to identify molecular targets that are selectively recognized by one of these peptides. These libraries have been successfully used to identify antibodies, pharmaceutical compounds, etc. Along these lines, phage libraries have been used for AD research to identify specific proteins present in AD patients but not in non‐AD.^[^ ^23^, ^24^, ^25^ ^]^ Using a novel, innovative approach, previously, we screened a phage library to identify phages whose peptides recognized antibodies against conformational‐specific A*β* species in the sera of AD patients.^[^ ^16^ ^]^ Here, we have analyzed six of these phages. Computational analysis indicates that the peptide sequences exposed by these phages recognize low molecular weight A*β* oligomers. It is tempting to speculate that, at least some of these six phages, recognize autoantibodies against these species of A*β*, which are widely reported as being highly toxic.^[^ ^9^, ^10^ ^]^

While the brain is an immunologically privileged organ, there is overwhelming evidence indicating the presence of circulating autoantibodies against toxic brain proteins, such as A*β*.^[^ ^11^, ^26^ ^]^ Since their identification, different groups have sought to determine whether these autoantibodies could be used as AD biomarkers. The results have not always been consistent, with some reports indicating that A*β* autoantibodies are lower in AD patients compared to healthy controls, others reporting high levels of A*β* autoantibodies in AD while some found no difference between the two groups (reviewed in ref. [27]). While these differences appear to be due to the method used to extract and analyze the autoantibodies, it is worthwhile noting that using ELISA measurement, Gruden and colleagues showed an increase in A*β*25‐35 oligomers in the sera of AD patients compared to healthy controls.^[^ ^28^ ^]^ This is notable as this middle region of A*β* is thought to be highly toxic,^[^ ^29^ ^]^ which is the region recognized by 12CIII1 and 12CIII3. Our results are consistent with these observations as they show that the signal obtained with each phage, which reflects the amount of circulating A*β* autoantibodies, increases as the disease progresses (Figure 4). Indeed, while the phages’ ability to recognize circulating A*β* autoantibodies is linked to the engineered exposed peptide, Figure 4 shows that as the MMSE decreases, the amount of signal for each phage increases. Thus, as the disease progresses, the change in signal for each individual phage is likely due to an increase in the circulating A*β* autoantibodies. The binding affinity between a peptide exposed by an individual phage and the A*β* autoantibodies would not change with the different stages of the disease.

A definite clinical diagnosis of AD is made *postmortem*, after a neuropathological confirmation of brain A*β* and tau accumulation. In 2011, the National Institute on Aging and the Alzheimer's association published general criteria for a general diagnosis of AD thus updating original guidelines published more than 25 years earlier.^[^ ^30^, ^31^ ^]^ One major difference in the updated version is the use of biomarkers to add to the clinical observation, to make a proper diagnosis of AD. Since its discovery, ^11^C‐labeled Pittsburg compound‐B has been used to identify amyloid deposits in patients’ brains following a PET scan.^[^ ^32^ ^]^ While this technique is widely used in clinical trials, there is an established dissociation between brain amyloid deposits and cognitive function.^[^ ^8^ ^]^ As such, there is an urgent unmet medical need for novel, non‐invasive, and accurate biomarkers.^[^ ^33^, ^34^ ^]^ Our results cater to this medical need as they identify how the combination of two phages, each exposing a unique peptide sequence on the capsid, can identify with 100% accuracy severe AD cases (MMSE < 12) and healthy controls (MMSE > 25), and with 90% accuracy mild‐to‐moderate AD case (MMSE between 12 and 24). Notably, our newly built algorithm can be refined with the addition of new cases analyzed, which may lead to an increase in its accuracy in identifying mild‐to‐moderate AD cases.

Not only can our system be used in clinical settings to aid in a diagnosis of AD, but it also has the potential to become a noninvasive and inexpensive surrogate endpoint biomarker to measure disease progression (or lack thereof) during clinical trials. Toward this end, an accurate biomarker to be used for this purpose may contribute to a reduction of sample size and/or duration of the trial itself therefore greatly diminishing overall costs.

## 4 Experimental Section

### Human Samples

Sera were obtained from the Neurologic Unit of the University Hospital “Policlinico Vittorio Emanuele” of Catania, Italy. AD diagnosis was done according to the criteria by the National Institute of Neurological and Communicative Disorders and Stroke and the Alzheimer's Disease and Related Disorders Association (NINCDS‐ADRDA).^[^ ^31^ ^]^ The severity of the disease was assessed by the MMSE. The study was approved by the Ethics Committee of the Policlinico Vittorio Emanuele of Catania, Italy.

### Phage‐Mediated Immuno‐PCR (PI‐PCR)

A 96‐well ELISA plate (Thermofisher) was coated with 200 µL per well solution of 0.5 µg mL^−1^ protein G in NaHCO_3_/NaCO_3_ coating buffer and the plates were incubated overnight at 4 °C. The plates were then washed with 250 µL per well of washing buffer (phosphate‐buffered saline (PBS) + 0.05% Tween20) after which 200 µL per well of sera diluted 1:50 was added and the plates were incubated for 1 h at 37 °C on an orbital shaker set to 100 rpm. The wells were then washed five times with washing buffer after which 300 µL per well of blocking buffer (Tris buffered saline + 5% w/v skimmed milk (TBSM)) was added and the plates were incubated at 37 °C for 2 h. After three washes with 250 µL per well of washing buffer, the phage preparations (10^11^ TU mL^−1^) were added and the plates were incubated for 1 h at 37 °C on an orbital shaker set to 100 rpm. To remove unbound phages, the wells were washed with TBS containing 5 × 10^−3^ m ethylenediaminetetraacetic acid and 0.1% Tween‐20. Subsequently, the wells were filled with 50 µL of ultrapure H_2_O and the plates were incubated in a water bath for 10 min at 95 °C to lyse the phages that were bound to the A*β* autoantibodies present in sera.

The real‐time PCRs were carried out using a biochip technology based on a silicon device integrating six microchambers.^[^ ^35^ ^]^ The reaction was performed using SsoAdvanced Universal SYBR Green (BioRad). The PCR premix consisted of 4 µL of DNA phage as a template, 5 µL of 2X Taq supermix, 0.2 × 10^−3^ m primer forward (5'GCTACCCTCGTTCCGATGCTGTC3') and reverse (5'GTTTTCCCAGTCACGAC3'). The step program for PCR was as follows: 94 °C for 5 min, followed by 30 cycles at 94 °C for 30 s; 52 °C for 30 s; 72 °C for 30 s. The optical capture phase was done in annealing.

### Quantitative Evaluation of Phage DNA and Standard Curves

To derive the amount of phage detected in PI‐PCR, a standard curve using known quantities of phages ranging from 10^2^ to 10^12^ was generated. An amplification curve and the relative Ct value were obtained for each concentration. All the Cts obtained to generate the standard curve were then plotted, which corresponded to the amount of phage DNA.

### Data Analysis

To normalize the amount of phage DNA to the input (amount of phage originally added to the wells), the following formula was used

(1) $$\%\;\mathrm{phage}\;\mathrm{bind}\;\mathrm{to}\;\mathrm{target}=\frac{\mathrm{phage}\;b\;\left(\frac{\mathrm{mg}}{\mathrm{ml}}\right)}{\mathrm{phage}\;i\;\left(\frac{\mathrm{mg}}{\mathrm{ml}}\right)}\times 100$$

where phage *b* is the quantity of phage bound to the target, and phage *i* is the quantity of phage clones initially added. The former was determined using the standard curve described above; the latter was determined by UV‐visible spectroscopy at 269 nm and used as absolute values. A standard absorbance of 0.38 was assumed to be equivalent to 0.1 mg mL^−1^. Thus, one unit of absorbance at 260 nm corresponds to 2.2 × 10^12^ TU mL^−1^.

### ELISA Test with Urea

Phage 12III1 in TBS (trisHCl/NaCl) at concentration of 10^11^ TU mL^−1^ was added in duplicate into 12 wells of 96‐well microtiter plate (Multisorp, Nunc, Roskilde, Denmark) at volume of 200 µL per well. Plates were incubated overnight at 4 °C. After which, they were incubated in blocking buffer (PBS−Tween 20 0.05–6% nonfat milk 300 µL per well) for 2 h at 37 °C and washed in PBS−0.05% Tween 20. The sera from 3 AD patients were prepared at dilution 1:50 (PBS+1% – nonfat milk – 0, 1% Tween20) and added in duplicate into the 12 wells (200 µL per well) and incubated 1 h at 37 °C while stirring. After the removal of the unbound sera, 250 µL per well of 6 m urea was added in 6 well, while the remaining 6 wells were incubated with PBS (control wells). Plates were incubated at 37 °C for 30 min while stirring. The plates were washed ten times with washing buffer (PBS−0.05% Tween 20) for 3 min each. After that the 12 wells were exposed to HRP‐conjugated anti‐human IgG (IgG Fc AP113P) diluted 1:15 000 in dilution buffer for 1 h at 37 °C while stirring. The plates were washed five times as above and developed with TMB for 30−45 min in the dark. After which, 100 µL of 1N HCl was added to stop the reaction. Optical absorbance at 450 nm (Labsystem Multiskan Bichromatic) was recorded.

### Computational Analysis

The phage‐peptides were analyzed for their potential binding to several assembly states of A*β* (from 1 to 12 monomers). The 3D structure of A*β* was obtained from RCSB‐Protein Data Back (https://www.rcsb.org), PDB ID: 2NAO. The 2NAO 3D structure was remodeled by us using YASARA software to build 3D structures of A*β* with 1, 3, 6, 9, and 12 monomers (called with alphabetic letters from A to N). Each performed 3D structure was used as “Enter PDB ID” in PepSurf (http://pepitope.tau.ac.il), while phage‐peptide were uploaded in Fasta format.^[^ ^19^ ^]^ The output of the program was the alignment of each phage‐peptide to the 3D structure. When several peptides were aligned, the server detected one or more patches of residues on the surface of the surveyed protein. Such a patch might correspond to a putative epitope site on the protein or a receptor‐binding site.^[^ ^36^ ^]^

### Discriminant Analysis

Discriminant Analysis (DA) was used to determine if results from phage clones could be used to discriminate the tested sera of healthy and diseased patients. Several data sets were initially built by using responses from a single phage clone or combining two or more phage clones. Phage responses were used as explanatory variables to identify the three clinical groups non‐AD, mild‐to‐moderate AD, and severe AD, pre‐assigned as qualitative dependent variables. Analyses were performed by using XLSTAT software, an Excel data analysis add‐on. Based on the data set, each serum was assigned factor scores, the probability to belong to a different clinical group, and squared Mahalanobis distances to the centroid of a clinical group. To visualize how each serum was discriminated for the pre‐assigned clinical groups, results were represented on 2D charts, including confidence ellipses and centroids. Finally, a confusion matrix summarized the reclassification of tested sera from each data set, allowing the identification of the overall percentage of well‐classified sera.

### Statistical Analysis

Data were analyzed using one or two‐way ANOVA followed by Bonferroni's correction unless otherwise stated. These analyses were performed using GraphPad Prism 8.

## Conflict of Interest

S.P.G., L.M.D., D.M., and S.C. hold a patent that covers Figures 1, 2, 3, 4. Title: Conformational Mimotopes for Detecting Specific Antibodies. Patent number: IT 102016000120204, WO2018096512 (A1), US2019383834.

## Author Contributions

M.G.R. performed the experimental study, L.M.D. performed the computational analyses, N.P. performed the experimental study, D.F. performed the discriminant analyses; M.N. performed the experimental study; E.L.S. performed the methodology; G.C. performed the experimental study, S.O. analyzed the data and wrote the manuscript, S.C. conceptualized the work, analyzed the data, and wrote the manuscript; S.P.G. conceptualized the work and analyzed the data.

## Supporting information

Supporting Information

## Data Availability

The data that support the findings of this study are available on request from the corresponding author. The data are not publicly available due to privacy or ethical restrictions.
