# Supplementary material for: A Novel Serum‐Based Diagnosis of Alzheimer's Disease Using an Advanced Phage‐Based Biochip
Source: Adv Sci (Weinh). 2023 May 7;10(21):2301650. doi: 10.1002/advs.202301650 (PMC10375152; doi:10.1002/advs.202301650)
Supplement: Supplementary file 1 — Supporting Information [file ADVS-10-2301650-s001.pdf]

ADVANCED SCIENCE
Open Access

Supporting Information

for *Adv. Sci.*, DOI 10.1002/advs.202301650

A Novel Serum-Based Diagnosis of Alzheimer's Disease Using an Advanced Phage-Based Biochip

Maria Giovanna Rizzo, Laura Maria De Plano, Nicoletta Palermo, Domenico Franco, Marco Nicolò, Emanuele Luigi Sciuto, Giovanna Calabrese, Salvatore Oddo, Sabrina Conoci* and Salvatore P. P. Guglielmino*

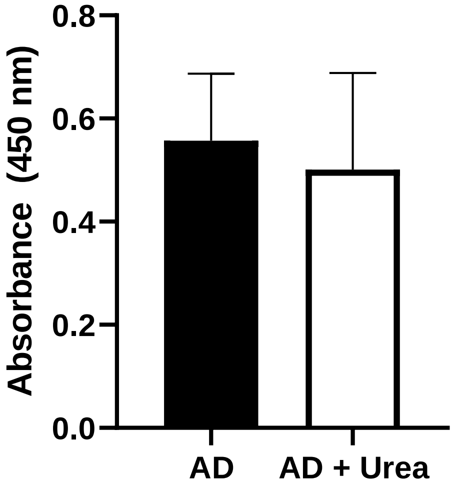

Figure S1. ELISA results for binding affinity of phage 12III1 to the IgG Aβ autoantibodies.

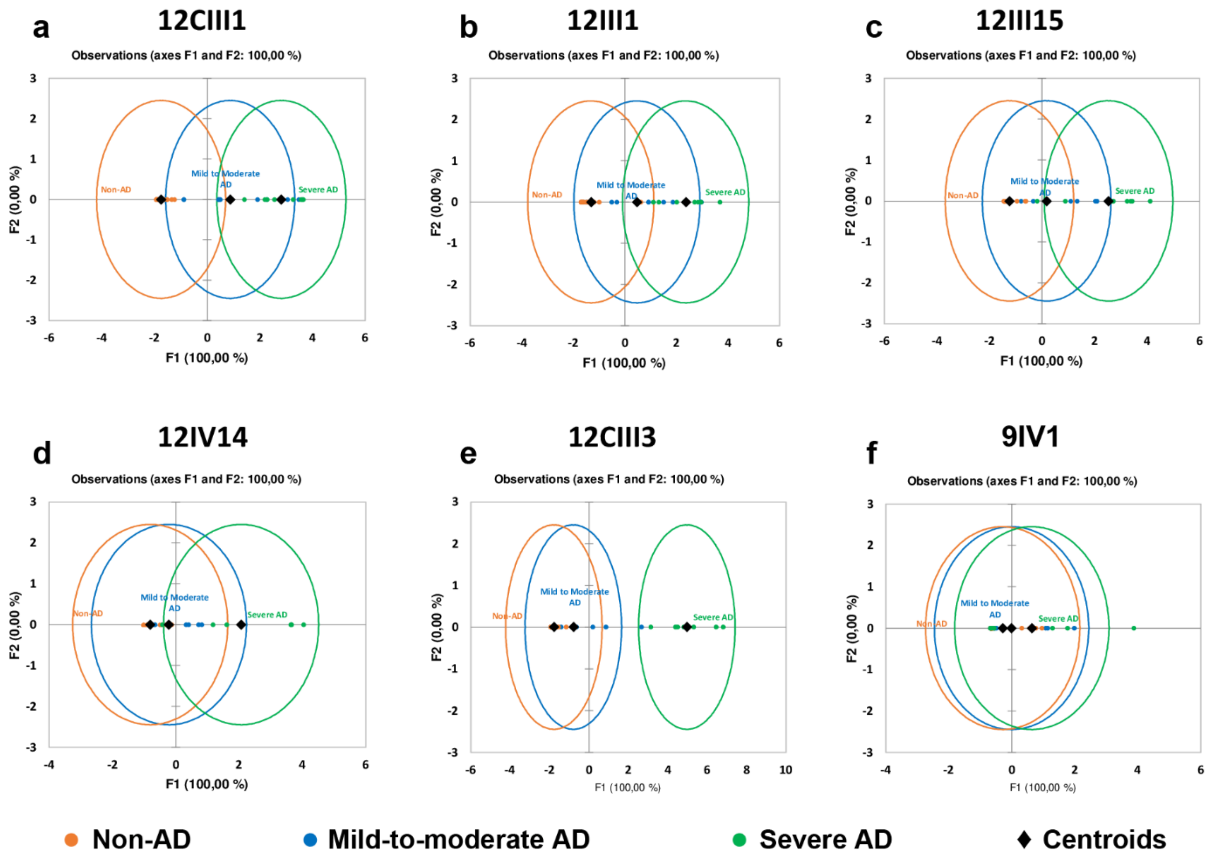

Figure S2. Discriminant analysis plot on a 1-dimensional map of sera by using a single phage clone. Orange, blue and green filled circles indicate sera identified as Non-AD, Mild-to-Moderate AD and Severe AD, respectively. The number and percentage of the sera correctly discriminated are summarized in the confusion matrix of Table 3. n=18 for Non-AD, 10 for Mild-to-Moderate AD and 8 for Severe AD.

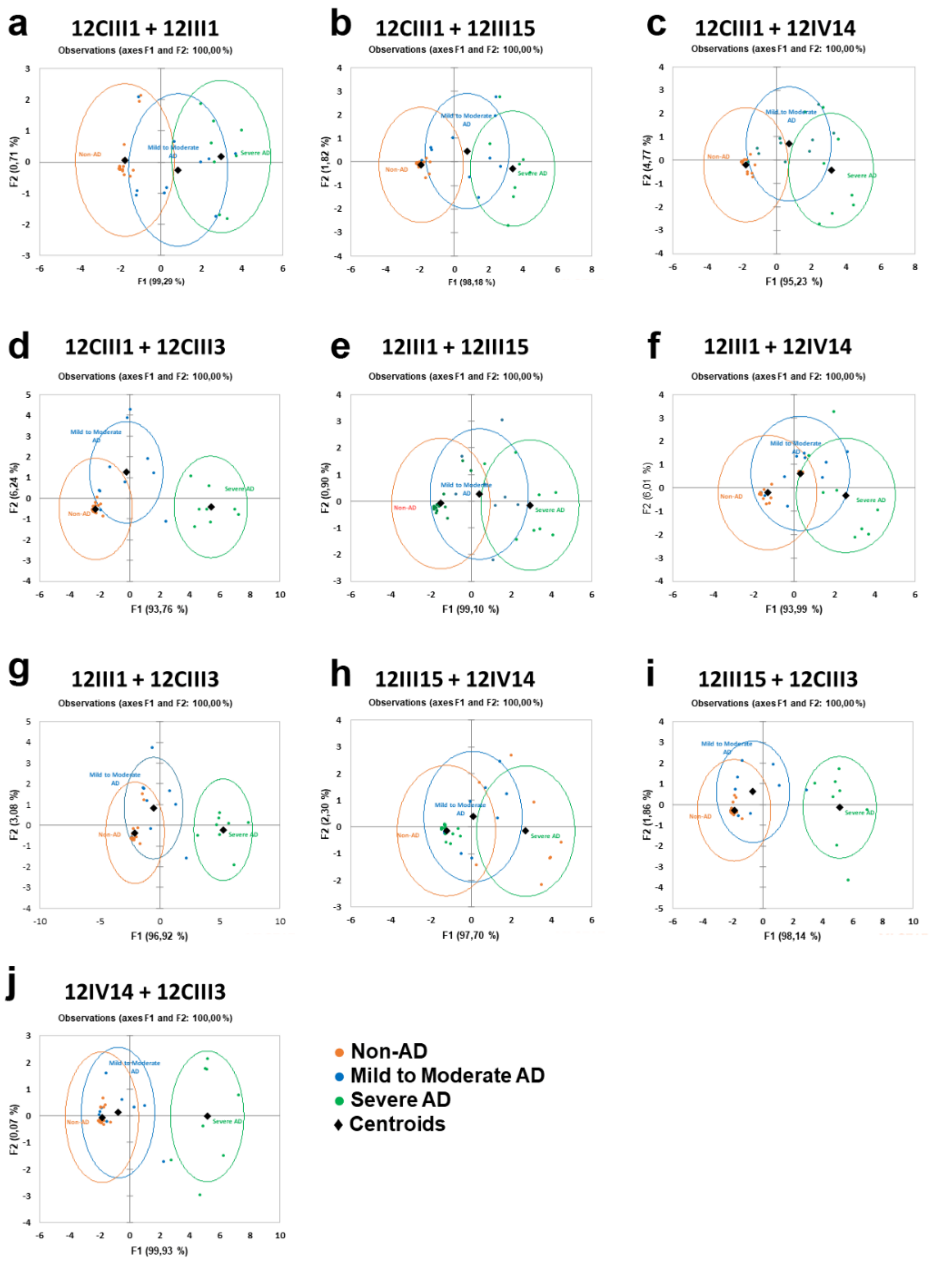

Figure S3. Discriminant analysis plot on a 2-dimensional map of 36 sera by using pairs of phage clones. Orange, blue and green filled circles indicate sera identified as Non-AD, Mild-to-Moderate AD and Severe AD, respectively. The number and percentage of the sera correctly discriminated are summarized in the confusion matrix of Table 4. n=18 for Non-AD, 10 for Mild-to-Moderate AD and 8 for Severe AD

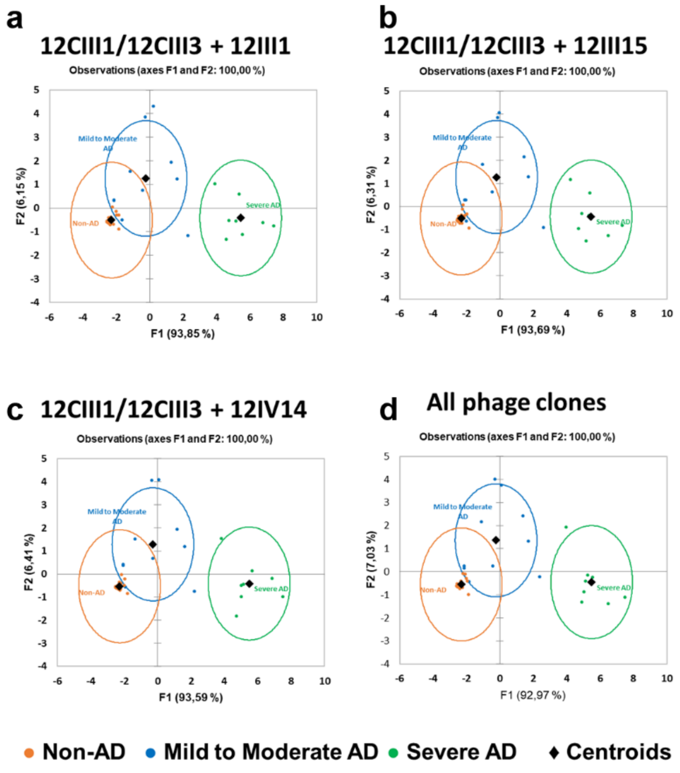

Figure S4. Discriminant analysis plot on a 2-dimensional map of 36 sera by using three or more clones. (a-c) The panels indicate the analyses done with the pair 12CIII1/12CIII3 and a third phage clone. (d) The panel indicates the analysis done including all phages. Orange, blue and green filled circles indicate sera identified as Non-AD, Mild-to-Moderate AD and Severe AD, respectively. The number and percentage of the sera correctly discriminated are summarized in the confusion matrix of Tables 5-6. n=18 for Non-AD, 10 for Mild-to-Moderate AD and 8 for Severe AD.

Table S1. **Homology prediction by Pepsurf analysis between 3D Amyloid-β structures and phage peptides.** he table shows on the top the amino acid primary sequence of Amyloid-β, and in the following rows the position of residues and chains mimicked by phage-peptide (green square).

	Aβ sequence																																									
	D	A	E	F	R	H	D	S	G	Y	E	V	H	H	Q	K	L	V	F	F	A	E	D	V	G	S	N	K	G	A	I	I	G	L	M	V	G	G	V	V	I	A
	12CIII1																																									
1																																	A	A		A	A	A				
3																																	A		A	A	A, B, C	A				
6									C		B	B			C	C																			F		F, E, D					
9																																	A, G	A		G	G	A, H				
12																																	F, N	N		F	F, N	N				
	12CIII3																																									
1																								A	A	A		A	A		A											
3																								A, C	A, B, C																	
6																																				A, C	A, B, C					
9																																				C	C	C, I	C			
	12III1																																									
1	A		A	A		A		A		A			A																													
3	C, B		C	C	C	C																																				
6	D		D	D	F	D, E		D		D																																
9	I		I	C, I	C, I	C, I																																				
12	N		N	N, F	F	N, F																																				
	12III15																																									
1				A	A	A	A	A	A	A	A																															
3					A, B	A, B	A, B		A	A																																
6					E, F	E, F	E		D	D, F	F																															
9				C	I	C, I			C, I	C, I	C	I																														
12				F	F, N	F, N	F		N	F, M	M																															
	12IV14																																									
1						A	A	A	A	A		A	A																													
3						B, C	C	B, C	B	B	B	B																														
6				B		B				A	A	A	A	B	A																						D, E					
9				F		F				F	F	F	F	F	F																						C	I				
12				F		F				F	F	N		F, N	N																						C	C				
	9IV1																																									
1																				A				A		A	A	A													A	
3					A, B		A	A, B		A																																
6				F	E, F		E	E, F		D																																
9								I	I	I		H		G		G, A		G																								
12						I		I		C		H		G		A, G		G																								

Table S2. Demographic characterization of sera

Clinical Group	Average age	Average MMSE	Sex
**Non-AD**	69	30	55,5 % M
**Mild-to-Moderate**	75	19	73 % M
**Severe**	75	9	25 % M

Table S3. Factor scores for clone 12CIII1. Probability of belonging to Non-AD, Mild-to-Moderate AD, Severe AD groups, and the squared Mahalanobis distances to the centroid of the group of tested 36 sera by using 12CIII1 phage clone. Incorrectly discriminated sera are in bold.

Sera	Prior	Posterior	Pr(Mild to moderate AD)	Pr(Severe AD)	Pr(Non-AD)	F1	D^2(Mild to moderate AD)	D^2(Severe AD)	D^2(Non-AD)
1	Non-AD	Non-AD	0.025	0.975	0.000	-1.902	-1.433	-8.789	27.551
2	Non-AD	Non-AD	0.022	0.978	0.000	-1.734	-1.731	-9.322	25.272
3	Non-AD	Non-AD	0.026	0.974	0.000	-1.925	-1.391	-8.623	27.867
4	Non-AD	Non-AD	0.022	0.978	0.000	-1.734	-1.731	-9.322	25.272
5	Non-AD	Non-AD	0.346	0.654	0.000	-1.233	-2.509	-3.781	18.981
6	Non-AD	Non-AD	0.026	0.974	0.000	-1.927	-1.388	-8.610	27.891
7	Non-AD	Non-AD	0.026	0.974	0.000	-1.921	-1.398	-8.653	27.814
8	Non-AD	Non-AD	0.025	0.975	0.000	-1.911	-1.417	-8.729	27.671
9	Non-AD	Non-AD	0.026	0.974	0.000	-1.916	-1.408	-8.693	27.739
10	Non-AD	Non-AD	0.023	0.977	0.000	-1.869	-1.492	-8.986	27.103
11	Non-AD	Non-AD	0.026	0.974	0.000	-1.927	-1.388	-8.609	27.893
12	Non-AD	Non-AD	0.046	0.954	0.000	-1.512	-2.096	-8.182	22.394
13	Non-AD	Non-AD	0.046	0.954	0.000	-1.512	-2.096	-8.182	22.394
14	Non-AD	Non-AD	0.154	0.846	0.000	-1.335	-2.364	-5.767	20.198
15	Non-AD	Non-AD	0.022	0.978	0.000	-1.740	-1.720	-9.324	25.361
16	Non-AD	Non-AD	0.021	0.979	0.000	-1.782	-1.648	-9.293	25.914
17	Non-AD	Non-AD	0.028	0.972	0.000	-1.626	-1.913	-9.030	23.855
18	Non-AD	Non-AD	0.023	0.977	0.000	-1.878	-1.476	-8.937	27.224
19	Mild to Moderate AD	Mild to Moderate AD	0.992	0.008	0.000	-0.877	-2.962	6.677	14.945
20	Mild to Moderate AD	Mild to Moderate AD	0.971	0.000	0.029	0.464	-3.920	94.457	3.092
21	Mild to Moderate AD	Mild to Moderate AD	0.992	0.008	0.000	-0.877	-2.962	6.677	14.945
22	Mild to Moderate AD	**Severe AD**	0.220	0.000	0.780	3.066	-2.408	483.199	-4.935
23	Mild to Moderate AD	**Severe AD**	0.212	0.000	0.788	3.491	-1.739	574.063	-4.369
24	Mild to Moderate AD	**Severe AD**	0.474	0.000	0.526	1.919	-3.623	276.212	-3.831
25	Mild to Moderate AD	**Non-AD**	0.031	0.969	0.000	-1.593	-1.966	-8.844	23.434
26	Mild to Moderate AD	Mild to Moderate AD	0.967	0.000	0.033	0.505	-3.930	98.288	2.816
27	Mild to Moderate AD	Mild to Moderate AD	0.973	0.000	0.027	0.440	-3.913	92.206	3.258
28	Mild to Moderate AD	**Severe AD**	0.350	0.000	0.650	2.271	-3.342	333.787	-4.579
29	Severe AD	Severe AD	0.216	0.000	0.784	3.615	-1.522	601.971	-4.105
30	Severe AD	Severe AD	0.214	0.000	0.786	3.570	-1.602	591.731	-4.206
31	Severe AD	Severe AD	0.218	0.000	0.782	3.660	-1.440	612.381	-3.997
32	Severe AD	Severe AD	0.212	0.000	0.788	3.264	-2.111	524.622	-4.737
33	Severe AD	Severe AD	0.371	0.000	0.629	2.203	-3.403	322.251	-4.462
34	Severe AD	Severe AD	0.281	0.000	0.719	2.565	-3.046	385.843	-4.925
35	Severe AD	**Mild to Moderate AD**	0.704	0.000	0.296	1.419	-3.881	203.679	-2.151
36	Severe AD	Severe AD	0.347	0.000	0.653	2.280	-3.333	335.418	-4.594

Table S4. Factor scores for clone 12III1. Probability of belonging to Non-AD, Mild-to-Moderate AD and Severe AD groups, and the squared Mahalanobis distances to the centroid of the group of tested 36 sera. Incorrectly discriminated sera are in bold.

Sera	Prior	Posterior	Pr(Mild to moderate AD)	Pr(Severe AD)	Pr(Non-AD)	F1	D²(Mild to moderate AD)	D²(Severe AD)	D²(Non-AD)
1	Non-AD	Non-AD	0.093	0.907	0.000	-1.704	-2.113	-6.661	17.056
2	Non-AD	Non-AD	0.095	0.905	0.000	-1.616	-2.287	-6.784	16.115
3	Non-AD	Non-AD	0.095	0.905	0.000	-1.650	-2.221	-6.741	16.475
4	Non-AD	Non-AD	0.101	0.899	0.000	-1.489	-2.527	-6.908	14.792
5	Non-AD	Non-AD	0.123	0.877	0.000	-1.202	-3.013	-6.944	11.963
6	Non-AD	Non-AD	0.095	0.905	0.000	-1.638	-2.244	-6.756	16.350
7	Non-AD	Non-AD	0.094	0.906	0.000	-1.660	-2.201	-6.726	16.584
8	Non-AD	Non-AD	0.094	0.906	0.000	-1.687	-2.147	-6.687	16.872
9	Non-AD	Non-AD	0.093	0.907	0.000	-1.706	-2.110	-6.658	17.072
10	Non-AD	Non-AD	0.094	0.906	0.000	-1.684	-2.154	-6.693	16.833
11	Non-AD	Non-AD	0.093	0.907	0.000	-1.706	-2.109	-6.658	17.075
12	Non-AD	**Mild to Moderate AD**	0.791	0.066	0.143	0.621	-4.309	0.644	-0.886
13	Non-AD	**Mild to Moderate AD**	0.784	0.122	0.094	0.456	-4.319	-0.600	-0.085
14	Non-AD	Non-AD	0.103	0.897	0.000	-1.440	-2.616	-6.938	14.292
15	Non-AD	Non-AD	0.097	0.903	0.000	-1.575	-2.367	-6.832	15.682
16	Non-AD	Non-AD	0.109	0.891	0.000	-1.348	-2.775	-6.967	13.378
17	Non-AD	Non-AD	0.095	0.905	0.000	-1.629	-2.263	-6.769	16.247
18	Non-AD	Non-AD	0.150	0.850	0.000	-1.003	-3.304	-6.773	10.137
19	Mild to Moderate AD	**Non-AD**	0.101	0.899	0.000	-1.489	-2.527	-6.908	14.792
20	Mild to Moderate AD	Mild to Moderate AD	0.728	0.019	0.253	0.910	-4.230	3.089	-2.114
21	Mild to Moderate AD	**Non-AD**	0.095	0.905	0.000	-1.616	-2.287	-6.784	16.115
22	Mild to Moderate AD	Mild to Moderate AD	0.728	0.019	0.253	0.910	-4.230	3.089	-2.114
23	Mild to Moderate AD	**Severe AD**	0.182	0.000	0.818	2.967	-1.431	30.277	-4.441
24	Mild to Moderate AD	**Severe AD**	0.488	0.001	0.511	1.499	-3.831	9.111	-3.926
25	Mild to Moderate AD	Mild to Moderate AD	0.791	0.094	0.115	0.530	-4.317	-0.055	-0.454
26	Mild to Moderate AD	**Non-AD**	0.387	0.608	0.005	-0.314	-4.030	-4.936	4.604
27	Mild to Moderate AD	**Non-AD**	0.291	0.707	0.002	-0.508	-3.870	-5.648	6.034
28	Mild to Moderate AD	**Severe AD**	0.361	0.000	0.639	1.854	-3.434	13.428	-4.575
29	Severe AD	Severe AD	0.177	0.000	0.823	3.692	0.494	43.962	-2.584
30	Severe AD	Severe AD	0.182	0.000	0.818	2.955	-1.459	30.063	-4.460
31	Severe AD	**Mild to Moderate AD**	0.564	0.002	0.434	1.317	-3.989	7.103	-3.465
32	Severe AD	**Mild to Moderate AD**	0.655	0.007	0.337	1.103	-4.135	4.904	-2.808
33	Severe AD	Severe AD	0.189	0.000	0.811	2.842	-1.713	28.133	-4.620
34	Severe AD	Severe AD	0.317	0.000	0.683	2.011	-3.222	15.497	-4.755
35	Severe AD	Severe AD	0.201	0.000	0.799	2.706	-2.004	25.872	-4.768
36	Severe AD	Severe AD	0.256	0.000	0.744	2.294	-2.779	19.490	-4.915

Table S5. Factor scores for clone 12III15. Probability to belong to Non-AD, Mild-to-Moderate AD and Severe AD groups, and the squared Mahalanobis distances to the centroid of the group of tested 36 sera. Incorrectly discriminated sera are in bold.

Sera	Prior	Posterior	Pr(Mild to moderate AD)	Pr(Severe AD)	Pr(Non-AD)	F1	D^2(Mild to moderate AD)	D^2(Severe AD)	D^2(Non-AD)
1	Non-AD	Non-AD	0.057	0.941	0.002	-1.233	-3.358	-8.966	2.895
2	Non-AD	Non-AD	0.057	0.941	0.002	-1.233	-3.358	-8.966	2.895
3	Non-AD	Non-AD	0.066	0.931	0.002	-1.437	-3.034	-8.321	3.644
4	Non-AD	Non-AD	0.058	0.939	0.002	-1.357	-3.165	-8.724	3.347
5	Non-AD	Non-AD	0.116	0.877	0.007	-0.953	-3.734	-7.772	1.929
6	Non-AD	Non-AD	0.064	0.934	0.002	-1.417	-3.067	-8.440	3.569
7	Non-AD	Non-AD	0.066	0.932	0.002	-1.434	-3.039	-8.341	3.632
8	Non-AD	Non-AD	0.067	0.930	0.002	-1.444	-3.021	-8.272	3.673
9	Non-AD	Non-AD	0.060	0.938	0.002	-1.381	-3.127	-8.626	3.434
10	Non-AD	Non-AD	0.063	0.934	0.002	-1.415	-3.070	-8.450	3.563
11	Non-AD	Non-AD	0.064	0.933	0.002	-1.422	-3.059	-8.413	3.587
12	Non-AD	**Mild to Moderate AD**	0.600	0.348	0.051	-0.609	-4.083	-2.994	0.842
13	Non-AD	Non-AD	0.061	0.936	0.003	-1.171	-3.448	-8.910	2.676
14	Non-AD	Non-AD	0.058	0.940	0.003	-1.218	-3.381	-8.963	2.839
15	Non-AD	Non-AD	0.056	0.941	0.002	-1.312	-3.238	-8.867	3.181
16	Non-AD	**Mild to Moderate AD**	0.548	0.407	0.045	-0.634	-4.062	-3.469	0.918
17	Non-AD	Non-AD	0.057	0.940	0.003	-1.223	-3.373	-8.965	2.860
18	Non-AD	Non-AD	0.056	0.942	0.002	-1.281	-3.286	-8.929	3.068
19	Mild to Moderate AD	**Non-AD**	0.058	0.939	0.002	-1.357	-3.165	-8.724	3.347
20	Mild to Moderate AD	Mild to Moderate AD	0.631	0.000	0.369	1.107	-3.967	75.098	-2.890
21	Mild to Moderate AD	**Non-AD**	0.057	0.941	0.002	-1.233	-3.358	-8.966	2.895
22	Mild to Moderate AD	Mild to Moderate AD	0.820	0.000	0.180	0.250	-4.411	24.774	-1.374
23	Mild to Moderate AD	Mild to Moderate AD	0.882	0.016	0.103	-0.321	-4.279	3.794	0.021
24	Mild to Moderate AD	Mild to Moderate AD	0.563	0.000	0.437	1.350	-3.700	93.501	-3.193
25	Mild to Moderate AD	**Non-AD**	0.056	0.941	0.002	-1.306	-3.247	-8.881	3.159
26	Mild to Moderate AD	**Severe AD**	0.343	0.000	0.657	2.104	-2.479	162.071	-3.776
27	Mild to Moderate AD	**Non-AD**	0.264	0.718	0.019	-0.787	-3.918	-5.921	1.390
28	Mild to Moderate AD	**Severe AD**	0.361	0.000	0.639	2.039	-2.609	155.430	-3.746
29	Severe AD	Severe AD	0.096	0.000	0.904	3.383	0.957	318.361	-3.530
30	Severe AD	Severe AD	0.196	0.000	0.804	2.724	-1.028	231.569	-3.850
31	Severe AD	**Mild to Moderate AD**	0.879	0.002	0.119	-0.183	-4.343	7.953	-0.345
32	Severe AD	Severe AD	0.203	0.000	0.797	2.690	-1.117	227.465	-3.856
33	Severe AD	**Mild to Moderate AD**	0.688	0.000	0.312	0.889	-4.153	60.166	-2.571
34	Severe AD	Severe AD	0.111	0.000	0.889	3.252	0.528	300.101	-3.626
35	Severe AD	Severe AD	0.039	0.000	0.961	4.124	3.731	431.825	-2.678
36	Severe AD	Severe AD	0.089	0.000	0.911	3.450	1.183	327.855	-3.475

Table S6. Factor scores for clone 12IV14. Probability of belonging to Non-AD, Mild-to-Moderate AD and Severe AD groups, and the squared Mahalanobis distances to the centroid of the group of tested 36 sera by using 12IV14 phage clone. Incorrectly discriminated sera are in bold.

Sera	Prior	Posterior	Pr(Mild to moderate AD)	Pr(Severe AD)	Pr(Non-AD)	F1	D^2(Mild to moderate AD)	D^2(Severe AD)	D^2(Non-AD)
1	Non-AD	Non-AD	0.134	0.844	0.022	-1.025	-4.319	-7.991	-0.681
2	Non-AD	Non-AD	0.132	0.848	0.021	-0.987	-4.442	-8.165	-0.742
3	Non-AD	Non-AD	0.133	0.846	0.021	-1.003	-4.391	-8.097	-0.717
4	Non-AD	Non-AD	0.268	0.696	0.036	-0.484	-5.507	-7.412	-1.473
5	Non-AD	Non-AD	0.268	0.696	0.036	-0.484	-5.507	-7.412	-1.473
6	Non-AD	Non-AD	0.134	0.845	0.022	-1.017	-4.344	-8.030	-0.694
7	Non-AD	Non-AD	0.133	0.846	0.021	-1.007	-4.377	-8.076	-0.710
8	Non-AD	Non-AD	0.134	0.844	0.022	-1.020	-4.335	-8.016	-0.689
9	Non-AD	Non-AD	0.134	0.845	0.021	-1.014	-4.356	-8.046	-0.699
10	Non-AD	Non-AD	0.133	0.845	0.021	-1.011	-4.364	-8.058	-0.703
11	Non-AD	Non-AD	0.133	0.845	0.021	-1.013	-4.359	-8.050	-0.701
12	Non-AD	**Mild to Moderate AD**	0.674	0.222	0.105	-0.124	-5.643	-3.418	-1.916
13	Non-AD	**Mild to Moderate AD**	0.468	0.466	0.066	-0.291	-5.645	-5.637	-1.718
14	Non-AD	Non-AD	0.132	0.847	0.021	-0.990	-4.430	-8.150	-0.737
15	Non-AD	Non-AD	0.149	0.831	0.020	-0.749	-5.071	-8.510	-1.103
16	Non-AD	Non-AD	0.331	0.624	0.045	-0.412	-5.576	-6.844	-1.567
17	Non-AD	Non-AD	0.130	0.850	0.020	-0.925	-4.626	-8.373	-0.838
18	Non-AD	Non-AD	0.131	0.849	0.019	-0.892	-4.721	-8.453	-0.890
19	Mild to Moderate AD	**Non-AD**	0.141	0.839	0.020	-0.791	-4.978	-8.539	-1.043
20	Mild to Moderate AD	**Non-AD**	0.268	0.696	0.036	-0.484	-5.507	-7.412	-1.473
21	Mild to Moderate AD	**Non-AD**	0.133	0.846	0.021	-1.003	-4.391	-8.097	-0.717
22	Mild to Moderate AD	**Non-AD**	0.171	0.806	0.023	-0.668	-5.235	-8.339	-1.221
23	Mild to Moderate AD	Mild to Moderate AD	0.587	0.000	0.413	0.833	-3.468	21.179	-2.766
24	Mild to Moderate AD	Mild to Moderate AD	0.643	0.000	0.357	0.731	-3.876	17.587	-2.698
25	Mild to Moderate AD	**Non-AD**	0.138	0.843	0.019	-0.815	-4.920	-8.539	-1.007
26	Mild to Moderate AD	**Non-AD**	0.177	0.799	0.024	-0.649	-5.269	-8.280	-1.247
27	Mild to Moderate AD	Mild to Moderate AD	0.790	0.003	0.207	0.334	-5.062	5.813	-2.382
28	Mild to Moderate AD	Mild to Moderate AD	0.769	0.001	0.229	0.414	-4.874	7.912	-2.453
29	Severe AD	Severe AD	0.000	0.000	1.000	3.645	24.268	211.182	-2.517
30	Severe AD	Severe AD	0.000	0.000	1.000	4.042	30.749	252.149	-2.152
31	Severe AD	Severe AD	0.357	0.000	0.643	1.178	-1.779	35.038	-2.956
32	Severe AD	**Non-AD**	0.325	0.631	0.044	-0.418	-5.570	-6.899	-1.559
33	Severe AD	**Non-AD**	0.136	0.845	0.019	-0.831	-4.881	-8.532	-0.983
34	Severe AD	Severe AD	0.110	0.000	0.890	1.618	1.071	56.550	-3.109
35	Severe AD	Severe AD	0.000	0.000	1.000	3.655	24.433	212.229	-2.508
36	Severe AD	Severe AD	0.000	0.000	1.000	3.655	24.433	212.229	-2.508

Table S7. Factor scores for clone 12CIII3. Probability to belonging to Non-AD, Mild-to-Moderate AD and Severe AD groups, and the squared Mahalanobis distances to the centroid of the group of tested 36 sera. Incorrectly discriminated sera are in bold.

Sera	Prior	Posterior	Pr(Mild to moderate AD)	Pr(Severe AD)	Pr(Non-AD)	F1	D^2(Mild to moderate AD)	D^2(Severe AD)	D^2(Non-AD)
1	Non-AD	Non-AD	0.070	0.930	0.000	-1.939	-4.589	-9.753	28.321
2	Non-AD	Non-AD	0.055	0.945	0.000	-1.746	-4.753	-10.454	26.471
3	Non-AD	Non-AD	0.063	0.937	0.000	-1.905	-4.620	-10.018	27.993
4	Non-AD	Non-AD	0.071	0.929	0.000	-1.941	-4.587	-9.738	28.337
5	Non-AD	Non-AD	0.071	0.929	0.000	-1.941	-4.587	-9.738	28.337
6	Non-AD	Non-AD	0.055	0.945	0.000	-1.832	-4.683	-10.384	27.289
7	Non-AD	Non-AD	0.056	0.944	0.000	-1.853	-4.666	-10.309	27.487
8	Non-AD	Non-AD	0.057	0.943	0.000	-1.714	-4.776	-10.382	26.176
9	Non-AD	Non-AD	0.054	0.946	0.000	-1.819	-4.695	-10.421	27.160
10	Non-AD	Non-AD	0.057	0.943	0.000	-1.864	-4.656	-10.260	27.594
11	Non-AD	Non-AD	0.067	0.933	0.000	-1.927	-4.600	-9.856	28.202
12	Non-AD	Non-AD	0.063	0.937	0.000	-1.905	-4.620	-10.022	27.987
13	Non-AD	Non-AD	0.068	0.932	0.000	-1.646	-4.825	-10.047	25.537
14	Non-AD	Non-AD	0.107	0.893	0.000	-1.547	-4.890	-9.123	24.622
15	Non-AD	**Mild to Moderate AD**	0.917	0.083	0.000	-1.150	-5.068	-0.269	21.102
16	Non-AD	Non-AD	0.054	0.946	0.000	-1.761	-4.741	-10.470	26.613
17	Non-AD	Non-AD	0.054	0.946	0.000	-1.815	-4.697	-10.429	27.127
18	Non-AD	Non-AD	0.075	0.925	0.000	-1.620	-4.843	-9.855	25.295
19	Mild to Moderate AD	**Non-AD**	0.070	0.930	0.000	-1.937	-4.591	-9.775	28.296
20	Mild to Moderate AD	**Non-AD**	0.056	0.944	0.000	-1.856	-4.663	-10.294	27.522
21	Mild to Moderate AD	**Non-AD**	0.063	0.937	0.000	-1.905	-4.620	-10.018	27.993
22	Mild to Moderate AD	**Non-AD**	0.071	0.929	0.000	-1.944	-4.585	-9.714	28.364
23	Mild to Moderate AD	**Non-AD**	0.067	0.933	0.000	-1.927	-4.600	-9.855	28.203
24	Mild to Moderate AD	Mild to Moderate AD	0.995	0.000	0.005	0.861	-4.023	171.749	6.655
25	Mild to Moderate AD	**Non-AD**	0.263	0.737	0.000	-1.411	-4.965	-7.030	23.396
26	Mild to Moderate AD	**Severe AD**	0.358	0.000	0.642	2.666	-0.315	506.814	-1.480
27	Mild to Moderate AD	Mild to Moderate AD	1.000	0.000	0.000	-0.642	-5.111	23.127	16.918
28	Mild to Moderate AD	Mild to Moderate AD	1.000	0.000	0.000	0.195	-4.729	91.287	10.809
29	Severe AD	Severe AD	0.000	0.000	1.000	6.807	18.100	1921.920	-2.885
30	Severe AD	Severe AD	0.004	0.000	0.996	4.426	5.827	998.356	-5.015
31	Severe AD	Severe AD	0.000	0.000	1.000	6.458	16.015	1767.883	-3.695
32	Severe AD	Severe AD	0.126	0.000	0.874	3.127	1.055	619.997	-2.828
33	Severe AD	Severe AD	0.001	0.000	0.999	5.312	9.863	1307.422	-5.151
34	Severe AD	Severe AD	0.002	0.000	0.998	4.815	7.520	1128.877	-5.211
35	Severe AD	Severe AD	0.004	0.000	0.996	4.493	6.108	1020.150	-5.064
36	Severe AD	Severe AD	0.005	0.000	0.995	4.389	5.670	986.235	-4.986

Table S8. Factor scores for clone 9IV1. Probability of belonging to Non-AD, Mild-to-Moderate AD and Severe AD groups, and the squared Mahalanobis distances to the centroid of the group of tested 36 sera. Incorrectly discriminated sera are in bold.

Sera	Prior	Posterior	Pr(Mild to moderate AD)	Pr(Severe AD)	Pr(Non-AD)	F1	D^2(Mild to moderate AD)	D^2(Severe AD)	D^2(Non-AD)
1	Non-AD	Non-AD	0.218	0.685	0.097	-0.675	-8.044	-10.337	-6.424
2	Non-AD	Non-AD	0.231	0.664	0.105	-0.024	-8.497	-10.606	-6.910
3	Non-AD	Non-AD	0.231	0.664	0.105	-0.024	-8.497	-10.606	-6.910
4	Non-AD	Non-AD	0.216	0.688	0.096	-0.650	-8.077	-10.396	-6.448
5	Non-AD	Non-AD	0.217	0.686	0.097	-0.668	-8.054	-10.354	-6.431
6	Non-AD	Non-AD	0.215	0.689	0.095	-0.644	-8.086	-10.411	-6.455
7	Non-AD	Non-AD	0.214	0.692	0.094	-0.615	-8.122	-10.474	-6.483
8	Non-AD	Non-AD	0.211	0.696	0.093	-0.575	-8.169	-10.552	-6.520
9	Non-AD	Non-AD	0.216	0.688	0.096	-0.658	-8.067	-10.378	-6.441
10	Non-AD	Non-AD	0.217	0.687	0.096	-0.659	-8.065	-10.375	-6.440
11	Non-AD	Non-AD	0.215	0.691	0.095	-0.632	-8.101	-10.437	-6.466
12	Non-AD	Non-AD	0.213	0.693	0.094	-0.604	-8.135	-10.496	-6.493
13	Non-AD	Non-AD	0.215	0.690	0.095	-0.635	-8.097	-10.430	-6.463
14	Non-AD	**Mild to Moderate AD**	0.415	0.312	0.274	0.770	-7.902	-7.331	-7.071
15	Non-AD	Non-AD	0.217	0.686	0.096	-0.666	-8.056	-10.358	-6.433
16	Non-AD	Non-AD	0.293	0.558	0.149	0.319	-8.394	-9.678	-7.038
17	Non-AD	**Mild to Moderate AD**	0.452	0.203	0.345	0.958	-7.577	-5.979	-7.039
18	Non-AD	Non-AD	0.361	0.429	0.209	0.573	-8.167	-8.513	-7.075
19	Mild to Moderate AD	**Non-AD**	0.218	0.685	0.097	-0.675	-8.045	-10.338	-6.424
20	Mild to Moderate AD	**Non-AD**	0.210	0.698	0.092	-0.548	-8.200	-10.599	-6.544
21	Mild to Moderate AD	**Non-AD**	0.208	0.701	0.091	-0.483	-8.266	-10.693	-6.600
22	Mild to Moderate AD	**Non-AD**	0.217	0.686	0.097	-0.670	-8.051	-10.348	-6.429
23	Mild to Moderate AD	**Non-AD**	0.216	0.688	0.096	-0.650	-8.077	-10.396	-6.448
24	Mild to Moderate AD	Mild to Moderate AD	0.464	0.118	0.418	1.141	-7.194	-4.453	-6.983
25	Mild to Moderate AD	**Severe AD**	0.284	0.002	0.714	1.987	-4.547	5.359	-6.394
26	Mild to Moderate AD	Mild to Moderate AD	0.463	0.142	0.395	1.082	-7.323	-4.963	-7.004
27	Mild to Moderate AD	**Non-AD**	0.210	0.699	0.092	-0.536	-8.212	-10.618	-6.555
28	Mild to Moderate AD	**Non-AD**	0.217	0.686	0.096	-0.666	-8.057	-10.359	-6.433
29	Severe AD	**Non-AD**	0.210	0.698	0.092	-0.551	-8.196	-10.594	-6.541
30	Severe AD	**Non-AD**	0.218	0.685	0.097	-0.675	-8.044	-10.336	-6.424
31	Severe AD	**Non-AD**	0.216	0.689	0.095	-0.644	-8.085	-10.410	-6.454
32	Severe AD	**Non-AD**	0.213	0.694	0.093	-0.598	-8.142	-10.507	-6.498
33	Severe AD	**Non-AD**	0.380	0.390	0.230	0.641	-8.085	-8.135	-7.077
34	Severe AD	Severe AD	0.455	0.067	0.478	1.295	-6.819	-3.002	-6.916
35	Severe AD	Severe AD	0.345	0.007	0.648	1.779	-5.331	2.529	-6.589
36	Severe AD	Severe AD	0.008	0.000	0.992	3.882	6.560	43.601	-3.117

Table S9. Factor scores for clones 12CIII1 and 12III1. Probability to belonging to Non-AD, Mild-to-Moderate AD and Severe AD groups, and the squared Mahalanobis distances to the centroid of the group of tested 36 sera. Incorrectly discriminated sera are in bold.

Sera	Prior	Posterior	Pr(Mild to moderate AD)	Pr(Severe AD)	Pr(Non-AD)	F1	F2	D^2(Mild to moderate AD)	D^2(Severe AD)	D^2(Non-AD)
1	Non-AD	Non-AD	0.016	0.984	0.000	-2.034	-0.248	-9.104	-17.372	48.675
2	Non-AD	Non-AD	0.016	0.984	0.000	-1.874	-0.299	-9.383	-17.686	45.054
3	Non-AD	Non-AD	0.017	0.983	0.000	-2.035	-0.163	-9.115	-17.251	48.353
4	Non-AD	Non-AD	0.015	0.985	0.000	-1.834	-0.150	-9.477	-17.859	43.521
5	Non-AD	Non-AD	0.393	0.607	0.000	-1.349	-0.274	-10.229	-11.097	33.254
6	Non-AD	Non-AD	0.017	0.983	0.000	-2.033	-0.148	-9.120	-17.241	48.234
7	Non-AD	Non-AD	0.017	0.983	0.000	-2.035	-0.179	-9.113	-17.276	48.420
8	Non-AD	Non-AD	0.016	0.984	0.000	-2.036	-0.220	-9.107	-17.332	48.596
9	Non-AD	Non-AD	0.016	0.984	0.000	-2.045	-0.237	-9.086	-17.290	48.901
10	Non-AD	Non-AD	0.015	0.985	0.000	-2.002	-0.254	-9.163	-17.544	47.919
11	Non-AD	Non-AD	0.017	0.983	0.000	-2.054	-0.227	-9.071	-17.216	49.077
12	Non-AD	Non-AD	0.129	0.871	0.000	-1.001	2.118	-6.415	-10.233	21.411
13	Non-AD	Non-AD	0.096	0.904	0.000	-1.053	1.924	-7.057	-11.548	22.459
14	Non-AD	Non-AD	0.194	0.806	0.000	-1.503	-0.460	-9.931	-12.781	37.295
15	Non-AD	Non-AD	0.015	0.985	0.000	-1.866	-0.245	-9.410	-17.768	44.651
16	Non-AD	Non-AD	0.014	0.986	0.000	-1.828	0.059	-9.461	-17.924	42.585
17	Non-AD	Non-AD	0.023	0.977	0.000	-1.792	-0.413	-9.482	-16.988	43.626
18	Non-AD	Non-AD	0.021	0.979	0.000	-1.797	0.551	-9.165	-16.829	40.212
19	Mild to Moderate AD	Mild to Moderate AD	0.999	0.001	0.000	-1.157	-0.939	-10.001	4.543	31.962
20	Mild to Moderate AD	Mild to Moderate AD	0.994	0.000	0.006	0.651	0.640	-11.141	90.321	-0.763
21	Mild to Moderate AD	Mild to Moderate AD	1.000	0.000	0.000	-1.196	-1.088	-9.736	5.649	33.459
22	Mild to Moderate AD	**Severe AD**	0.366	0.000	0.634	2.706	-1.753	-9.046	554.027	-10.148
23	Mild to Moderate AD	**Severe AD**	0.194	0.000	0.806	3.683	0.266	-8.834	609.722	-11.683
24	Mild to Moderate AD	Mild to Moderate AD	0.600	0.000	0.400	1.983	-0.008	-11.302	292.124	-10.490
25	Mild to Moderate AD	**Non-AD**	0.113	0.887	0.000	-1.094	2.086	-6.437	-10.564	23.083
26	Mild to Moderate AD	Mild to Moderate AD	1.000	0.000	0.000	0.301	-0.832	-11.401	107.561	6.684
27	Mild to Moderate AD	Mild to Moderate AD	1.000	0.000	0.000	0.189	-1.000	-11.161	102.766	8.791
28	Mild to Moderate AD	**Severe AD**	0.358	0.000	0.642	2.372	0.084	-10.901	353.163	-12.072
29	Severe AD	Severe AD	0.232	0.000	0.768	4.007	1.002	-6.929	626.870	-9.319
30	Severe AD	Severe AD	0.205	0.000	0.795	3.741	0.179	-8.811	630.444	-11.525
31	Severe AD	Severe AD	0.212	0.000	0.788	3.303	-1.822	-8.118	699.768	-10.738
32	Severe AD	Severe AD	0.287	0.000	0.713	2.923	-1.710	-8.892	599.099	-10.717
33	Severe AD	Severe AD	0.139	0.000	0.861	2.626	1.304	-8.546	325.842	-12.189
34	Severe AD	Severe AD	0.266	0.000	0.734	2.653	-0.002	-10.643	410.880	-12.669
35	Severe AD	Severe AD	0.219	0.000	0.781	1.965	1.865	-7.508	197.848	-10.056
36	Severe AD	Severe AD	0.238	0.000	0.762	2.517	0.591	-10.186	347.911	-12.512

Table S10. Factor scores for clones 12CIII1 and 12III15. Probability to belonging to Non-AD, Mild-to-Moderate AD and Severe AD groups, and the squared Mahalanobis distances to the centroid of the group of tested 36 sera. Incorrectly discriminated sera are in bold.

Sera	Prior	Posterior	Pr(Mild to moderate AD)	Pr(Severe AD)	Pr(Non-AD)	F1	F2	D^2(Mild to moderate AD)	D^2(Severe AD)	D^2(Non-AD)
1	Non-AD	Non-AD	0.005	0.995	0.000	-2.047	-0.237	-8.624	-19.275	48.027
2	Non-AD	Non-AD	0.004	0.996	0.000	-1.921	-0.118	-8.890	-20.031	44.851
3	Non-AD	Non-AD	0.005	0.995	0.000	-2.167	-0.070	-8.469	-19.165	50.091
4	Non-AD	Non-AD	0.004	0.996	0.000	-1.984	-0.006	-8.812	-19.650	45.818
5	Non-AD	Non-AD	0.086	0.914	0.000	-1.405	-0.016	-9.707	-14.434	33.967
6	Non-AD	Non-AD	0.005	0.995	0.000	-2.158	-0.090	-8.480	-19.223	49.963
7	Non-AD	Non-AD	0.005	0.995	0.000	-2.162	-0.071	-8.477	-19.197	49.989
8	Non-AD	Non-AD	0.005	0.995	0.000	-2.160	-0.053	-8.485	-19.199	49.877
9	Non-AD	Non-AD	0.004	0.996	0.000	-2.132	-0.114	-8.522	-19.373	49.458
10	Non-AD	Non-AD	0.004	0.996	0.000	-2.114	-0.051	-8.570	-19.468	48.846
11	Non-AD	Non-AD	0.005	0.995	0.000	-2.161	-0.085	-8.477	-19.207	50.004
12	Non-AD	Non-AD	0.091	0.909	0.000	-1.440	-0.523	-9.342	-13.956	36.313
13	Non-AD	Non-AD	0.009	0.991	0.000	-1.724	-0.017	-9.240	-18.718	40.347
14	Non-AD	Non-AD	0.055	0.945	0.000	-1.614	0.150	-9.432	-15.106	37.592
15	Non-AD	Non-AD	0.004	0.996	0.000	-1.966	-0.052	-8.833	-19.885	45.587
16	Non-AD	Non-AD	0.214	0.786	0.000	-1.655	-0.691	-8.872	-11.475	41.297
17	Non-AD	Non-AD	0.005	0.995	0.000	-1.835	-0.051	-9.053	-19.640	42.791
18	Non-AD	Non-AD	0.004	0.996	0.000	-2.053	-0.177	-8.640	-19.616	47.948
19	Mild to Moderate AD	Mild to Moderate AD	1.000	0.000	0.000	-1.341	0.601	-9.721	6.300	31.069
20	Mild to Moderate AD	Mild to Moderate AD	0.997	0.000	0.003	0.906	-0.667	-10.356	102.067	1.258
21	Mild to Moderate AD	Mild to Moderate AD	0.998	0.002	0.000	-1.278	0.489	-9.858	2.897	30.184
22	Mild to Moderate AD	**Severe AD**	0.472	0.000	0.528	2.424	1.946	-9.226	530.558	-9.448
23	Mild to Moderate AD	**Severe AD**	0.399	0.000	0.601	2.455	2.761	-7.352	697.509	-8.173
24	Mild to Moderate AD	Mild to Moderate AD	0.674	0.000	0.326	2.118	0.144	-10.485	266.534	-9.037
25	Mild to Moderate AD	**Non-AD**	0.007	0.993	0.000	-1.852	0.047	-9.044	-18.910	42.833
26	Mild to Moderate AD	Mild to Moderate AD	0.958	0.000	0.042	1.438	-1.536	-8.201	165.625	-1.930
27	Mild to Moderate AD	Mild to Moderate AD	1.000	0.000	0.000	-0.066	1.020	-10.730	101.993	9.990
28	Mild to Moderate AD	**Severe AD**	0.292	0.000	0.708	2.729	-0.226	-9.413	333.175	-11.185
29	Severe AD	Severe AD	0.099	0.000	0.901	4.414	-0.484	-5.645	620.547	-10.071
30	Severe AD	Severe AD	0.140	0.000	0.860	4.048	0.077	-7.337	587.064	-10.976
31	Severe AD	Severe AD	0.349	0.000	0.651	2.652	2.756	-7.202	732.724	-8.448
32	Severe AD	Severe AD	0.119	0.000	0.881	3.802	-0.109	-7.661	524.308	-11.662
33	Severe AD	**Mild to Moderate AD**	0.685	0.000	0.315	2.099	0.760	-10.641	315.571	-9.088
34	Severe AD	Severe AD	0.059	0.000	0.941	3.561	-1.110	-6.270	438.817	-11.805
35	Severe AD	Severe AD	0.023	0.000	0.977	3.141	-2.705	-1.434	434.269	-8.974
36	Severe AD	Severe AD	0.047	0.000	0.953	3.447	-1.489	-5.451	420.067	-11.472

Table S11. Factor scores for clones 12CIII1 and 12IV14. Probability to belonging to Non-AD, Mild-to-Moderate AD and Severe AD groups, and the squared Mahalanobis distances to the centroid of the group of tested 36 sera. Incorrectly discriminated sera are in bold.

Sera	Prior	Posterior	Pr(Mild to moderate AD)	Pr(Severe AD)	Pr(Non-AD)	F1	F2	D^2(Mild to moderate AD)	D^2(Severe AD)	D^2(Non-AD)
1	Non-AD	Non-AD	0.013	0.987	0.000	-1.990	-0.064	-10.261	-18.954	24.352
2	Non-AD	Non-AD	0.014	0.986	0.000	-1.832	-0.008	-10.556	-19.038	21.988
3	Non-AD	Non-AD	0.013	0.987	0.000	-2.003	-0.099	-10.228	-18.891	24.631
4	Non-AD	Non-AD	0.016	0.984	0.000	-1.663	-0.497	-9.836	-18.049	21.183
5	Non-AD	Non-AD	0.183	0.817	0.000	-1.230	-0.218	-10.998	-13.985	14.847
6	Non-AD	Non-AD	0.013	0.987	0.000	-2.009	-0.086	-10.221	-18.850	24.681
7	Non-AD	Non-AD	0.013	0.987	0.000	-2.001	-0.092	-10.234	-18.905	24.585
8	Non-AD	Non-AD	0.013	0.987	0.000	-1.996	-0.074	-10.248	-18.929	24.464
9	Non-AD	Non-AD	0.013	0.987	0.000	-1.998	-0.083	-10.242	-18.921	24.520
10	Non-AD	Non-AD	0.012	0.988	0.000	-1.957	-0.060	-10.323	-19.111	23.876
11	Non-AD	Non-AD	0.013	0.987	0.000	-2.008	-0.090	-10.222	-18.859	24.675
12	Non-AD	Non-AD	0.058	0.942	0.000	-1.350	-0.724	-8.875	-14.449	17.791
13	Non-AD	Non-AD	0.029	0.971	0.000	-1.406	-0.561	-9.701	-16.726	18.013
14	Non-AD	Non-AD	0.412	0.588	0.000	-1.488	0.218	-11.091	-11.800	16.877
15	Non-AD	Non-AD	0.010	0.990	0.000	-1.758	-0.242	-10.439	-19.602	21.681
16	Non-AD	Non-AD	0.026	0.974	0.000	-1.680	-0.594	-9.444	-16.695	21.724
17	Non-AD	Non-AD	0.019	0.981	0.000	-1.718	-0.007	-10.748	-18.585	20.454
18	Non-AD	Non-AD	0.011	0.989	0.000	-1.925	-0.181	-10.294	-19.253	23.793
19	Mild to Moderate AD	Mild to Moderate AD	1.000	0.000	0.000	-1.024	0.280	-11.764	3.796	11.246
20	Mild to Moderate AD	Mild to Moderate AD	0.997	0.000	0.003	0.239	0.730	-12.662	117.522	-1.192
21	Mild to Moderate AD	Mild to Moderate AD	1.000	0.000	0.000	-1.096	0.486	-11.439	9.230	11.597
22	Mild to Moderate AD	**Severe AD**	0.257	0.000	0.743	2.428	2.362	-7.065	693.664	-9.185
23	Mild to Moderate AD	Mild to Moderate AD	0.501	0.000	0.499	3.300	1.138	-10.178	666.358	-10.169
24	Mild to Moderate AD	Mild to Moderate AD	0.663	0.000	0.337	1.906	0.360	-10.852	296.222	-9.498
25	Mild to Moderate AD	**Non-AD**	0.017	0.983	0.000	-1.653	-0.097	-10.784	-18.884	19.846
26	Mild to Moderate AD	Mild to Moderate AD	0.996	0.000	0.004	0.218	0.914	-12.345	131.248	-1.232
27	Mild to Moderate AD	Mild to Moderate AD	0.990	0.000	0.010	0.493	-0.079	-11.100	87.562	-1.995
28	Mild to Moderate AD	Mild to Moderate AD	0.726	0.000	0.274	2.104	0.864	-11.960	386.198	-10.010
29	Severe AD	Severe AD	0.000	0.000	1.000	4.353	-1.529	25.681	592.427	-9.590
30	Severe AD	Severe AD	0.000	0.000	1.000	4.448	-1.940	35.729	588.361	-9.318
31	Severe AD	Severe AD	0.358	0.000	0.642	3.563	0.897	-8.815	688.032	-9.985
32	Severe AD	Severe AD	0.358	0.000	0.642	2.683	2.229	-8.170	723.982	-9.338
33	Severe AD	**Mild to Moderate AD**	0.529	0.000	0.471	1.626	2.038	-8.623	474.604	-8.395
34	Severe AD	Severe AD	0.045	0.000	0.955	2.763	-0.143	-4.908	391.345	-11.039
35	Severe AD	Severe AD	0.000	0.000	1.000	2.458	-2.765	38.948	256.662	-7.736
36	Severe AD	Severe AD	0.000	0.000	1.000	3.202	-2.284	33.141	352.124	-10.138

Table S12. Factor scores for clones 12CIII1 and 12CIII3. Probability to belonging to Non-AD, Mild-to-Moderate AD and Severe AD groups, and the squared Mahalanobis distances to the centroid of the group of tested 36 sera. Incorrectly discriminated sera are in bold.

Sera	Prior	Posterior	Pr(Mild to moderate AD)	Pr(Severe AD)	Pr(Non-AD)	F1	F2	D^2(Mild to moderate AD)	D^2(Severe AD)	D^2(Non-AD)
1	Non-AD	Non-AD	0.003	0.997	0.000	-2.485	-0.555	-8.775	-20.088	36.289
2	Non-AD	Non-AD	0.002	0.998	0.000	-2.247	-0.519	-9.195	-21.180	33.191
3	Non-AD	Non-AD	0.003	0.997	0.000	-2.468	-0.595	-8.759	-20.174	36.278
4	Non-AD	Non-AD	0.003	0.997	0.000	-2.407	-0.404	-9.061	-20.441	34.618
5	Non-AD	Non-AD	0.096	0.904	0.000	-2.171	0.043	-9.807	-14.283	30.160
6	Non-AD	Non-AD	0.003	0.997	0.000	-2.408	-0.640	-8.807	-20.450	35.767
7	Non-AD	Non-AD	0.003	0.997	0.000	-2.423	-0.623	-8.803	-20.439	35.857
8	Non-AD	Non-AD	0.003	0.997	0.000	-2.304	-0.695	-8.908	-20.440	34.770
9	Non-AD	Non-AD	0.003	0.997	0.000	-2.392	-0.638	-8.835	-20.554	35.555
10	Non-AD	Non-AD	0.003	0.997	0.000	-2.407	-0.570	-8.886	-20.711	35.405
11	Non-AD	Non-AD	0.004	0.996	0.000	-2.486	-0.584	-8.740	-20.021	36.454
12	Non-AD	Non-AD	0.007	0.993	0.000	-2.273	-0.227	-9.440	-19.391	32.265
13	Non-AD	Non-AD	0.006	0.994	0.000	-2.061	-0.380	-9.607	-19.790	30.357
14	Non-AD	Non-AD	0.031	0.969	0.000	-1.896	-0.280	-9.918	-16.788	28.058
15	Non-AD	Non-AD	0.328	0.672	0.000	-1.762	-0.876	-9.401	-10.834	29.273
16	Non-AD	Non-AD	0.002	0.998	0.000	-2.282	-0.553	-9.105	-21.162	33.776
17	Non-AD	Non-AD	0.003	0.997	0.000	-2.253	-0.382	-9.327	-20.825	32.651
18	Non-AD	Non-AD	0.004	0.996	0.000	-2.212	-0.722	-9.016	-20.049	33.777
19	Mild to Moderate AD	Mild to Moderate AD	0.969	0.031	0.000	-2.000	0.360	-10.241	-3.335	27.450
20	Mild to Moderate AD	Mild to Moderate AD	1.000	0.000	0.000	-1.303	1.510	-11.177	85.677	20.392
21	Mild to Moderate AD	Mild to Moderate AD	0.963	0.037	0.000	-1.974	0.341	-10.267	-3.751	27.197
22	Mild to Moderate AD	Mild to Moderate AD	1.000	0.000	0.000	-0.149	3.886	-9.383	485.611	25.735
23	Mild to Moderate AD	Mild to Moderate AD	1.000	0.000	0.000	0.064	4.255	-8.695	577.793	28.397
24	Mild to Moderate AD	Mild to Moderate AD	0.988	0.000	0.012	1.609	1.206	-10.315	394.405	-1.489
25	Mild to Moderate AD	**Non-AD**	0.018	0.982	0.000	-1.907	-0.591	-9.583	-17.549	29.495
26	Mild to Moderate AD	Mild to Moderate AD	0.759	0.000	0.241	2.422	-1.122	-6.693	552.167	-4.395
27	Mild to Moderate AD	Mild to Moderate AD	1.000	0.000	0.000	-0.319	0.772	-11.589	100.839	10.722
28	Mild to Moderate AD	Mild to Moderate AD	0.999	0.000	0.001	1.229	1.913	-10.722	382.003	3.155
29	Severe AD	Severe AD	0.000	0.000	1.000	7.279	-0.787	12.866	2279.374	-10.804
30	Severe AD	Severe AD	0.002	0.000	0.998	5.307	0.578	0.852	1410.870	-11.908
31	Severe AD	Severe AD	0.000	0.000	1.000	7.015	-0.540	10.888	2142.622	-11.434
32	Severe AD	Severe AD	0.058	0.000	0.942	4.099	1.071	-4.167	1010.126	-9.740
33	Severe AD	Severe AD	0.000	0.000	1.000	5.390	-1.166	3.491	1482.153	-12.503
34	Severe AD	Severe AD	0.001	0.000	0.999	5.153	-0.550	1.400	1362.613	-13.278
35	Severe AD	Severe AD	0.007	0.000	0.993	4.350	-1.383	-0.438	1120.135	-10.478
36	Severe AD	Severe AD	0.002	0.000	0.998	4.670	-0.552	-0.612	1188.610	-12.862

Table S13. Factor scores for clones 12III1 and 12III15. Probability to belonging to Non-AD, Mild-to-Moderate AD and Severe AD groups, and the squared Mahalanobis distances to the centroid of the group of tested 36 sera. Incorrectly discriminated sera are in bold.

Sera	Prior	Posterior	Pr(Mild to moderate AD)	Pr(Severe AD)	Pr(Non-AD)	F1	F2	D^2(Mild to moderate AD)	D^2(Severe AD)	D^2(Non-AD)
1	Non-AD	Non-AD	0.017	0.983	0.000	-1.749	-0.443	-9.162	-17.292	10.836
2	Non-AD	Non-AD	0.017	0.983	0.000	-1.697	-0.362	-9.311	-17.484	9.971
3	Non-AD	Non-AD	0.018	0.982	0.000	-1.839	-0.207	-9.090	-17.123	10.604
4	Non-AD	Non-AD	0.017	0.983	0.000	-1.696	-0.131	-9.406	-17.523	8.957
5	Non-AD	Non-AD	0.045	0.955	0.000	-1.283	-0.236	-10.102	-16.191	5.792
6	Non-AD	Non-AD	0.017	0.983	0.000	-1.820	-0.215	-9.127	-17.242	10.459
7	Non-AD	Non-AD	0.017	0.983	0.000	-1.843	-0.219	-9.077	-17.143	10.698
8	Non-AD	Non-AD	0.018	0.982	0.000	-1.866	-0.234	-9.024	-17.073	10.976
9	Non-AD	Non-AD	0.015	0.985	0.000	-1.838	-0.310	-9.050	-17.361	11.059
10	Non-AD	Non-AD	0.016	0.984	0.000	-1.846	-0.258	-9.057	-17.238	10.895
11	Non-AD	Non-AD	0.016	0.984	0.000	-1.863	-0.272	-9.014	-17.196	11.124
12	Non-AD	**Mild to Moderate AD**	0.683	0.272	0.045	0.001	1.129	-11.031	-9.187	-5.588
13	Non-AD	**Mild to Moderate AD**	0.661	0.316	0.023	-0.436	1.491	-10.323	-8.844	-3.630
14	Non-AD	Non-AD	0.017	0.983	0.000	-1.583	-0.214	-9.596	-17.695	8.286
15	Non-AD	Non-AD	0.016	0.984	0.000	-1.720	-0.252	-9.318	-17.618	9.691
16	Non-AD	**Mild to Moderate AD**	0.625	0.375	0.000	-1.178	-0.663	-9.980	-8.958	6.735
17	Non-AD	Non-AD	0.017	0.983	0.000	-1.698	-0.382	-9.298	-17.425	10.078
18	Non-AD	Non-AD	0.027	0.973	0.000	-1.364	0.247	-10.050	-17.243	4.705
19	Mild to Moderate AD	**Non-AD**	0.017	0.983	0.000	-1.696	-0.131	-9.406	-17.523	8.957
20	Mild to Moderate AD	Mild to Moderate AD	0.739	0.000	0.261	1.204	-0.173	-11.078	74.811	-8.994
21	Mild to Moderate AD	**Non-AD**	0.017	0.983	0.000	-1.697	-0.362	-9.311	-17.484	9.971
22	Mild to Moderate AD	Mild to Moderate AD	0.841	0.000	0.159	0.688	0.611	-11.427	16.127	-8.097
23	Mild to Moderate AD	Mild to Moderate AD	0.663	0.000	0.337	1.559	3.028	-7.006	19.491	-5.655
24	Mild to Moderate AD	**Severe AD**	0.495	0.000	0.505	1.697	0.147	-10.687	91.385	-10.724
25	Mild to Moderate AD	Mild to Moderate AD	0.879	0.087	0.034	-0.473	1.683	-10.007	-5.385	-3.477
26	Mild to Moderate AD	Mild to Moderate AD	0.959	0.000	0.041	1.081	-2.214	-7.869	217.851	-1.556
27	Mild to Moderate AD	**Non-AD**	0.128	0.871	0.000	-0.774	0.251	-10.875	-14.708	0.278
28	Mild to Moderate AD	**Severe AD**	0.269	0.000	0.731	2.322	-0.156	-9.583	162.463	-11.585
29	Severe AD	Severe AD	0.072	0.000	0.928	4.216	0.309	-4.513	331.275	-9.612
30	Severe AD	Severe AD	0.107	0.000	0.893	3.384	0.232	-7.217	239.049	-11.468
31	Severe AD	**Mild to Moderate AD**	0.794	0.001	0.205	0.668	1.381	-10.845	1.719	-8.137
32	Severe AD	Severe AD	0.342	0.000	0.658	2.270	-1.444	-8.032	271.027	-9.341
33	Severe AD	Severe AD	0.416	0.000	0.584	2.213	1.806	-8.857	52.723	-9.537
34	Severe AD	Severe AD	0.086	0.000	0.914	3.145	-1.121	-6.582	344.193	-11.300
35	Severe AD	Severe AD	0.024	0.000	0.976	4.079	-1.277	-3.316	496.943	-10.761
36	Severe AD	Severe AD	0.059	0.000	0.941	3.430	-1.040	-5.894	373.040	-11.440

Table S14. Factor scores for clones 12III1 and 12IV14. Probability to belonging to Non-AD, Mild-to-Moderate AD and Severe AD groups, and the squared Mahalanobis distances to the centroid of the group of tested 36 sera. Incorrectly discriminated sera are in bold.

Sera	Prior	Posterior	Pr(Mild to moderate AD)	Pr(Severe AD)	Pr(Non-AD)	F1	F2	D^2(Mild to moderate AD)	D^2(Severe AD)	D^2(Non-AD)
1	Non-AD	Non-AD	0.040	0.960	0.000	-1.696	-0.277	-11.041	-17.386	11.933
2	Non-AD	Non-AD	0.040	0.960	0.000	-1.613	-0.247	-11.220	-17.553	10.893
3	Non-AD	Non-AD	0.040	0.960	0.000	-1.646	-0.257	-11.151	-17.486	11.289
4	Non-AD	Non-AD	0.218	0.782	0.000	-1.346	-0.694	-10.796	-13.351	10.093
5	Non-AD	Non-AD	0.126	0.874	0.000	-1.117	-0.458	-11.573	-15.442	6.725
6	Non-AD	Non-AD	0.043	0.957	0.000	-1.641	-0.231	-11.164	-17.392	11.123
7	Non-AD	Non-AD	0.040	0.960	0.000	-1.655	-0.261	-11.130	-17.466	11.410
8	Non-AD	Non-AD	0.040	0.960	0.000	-1.681	-0.269	-11.075	-17.407	11.727
9	Non-AD	Non-AD	0.039	0.961	0.000	-1.694	-0.291	-11.044	-17.445	11.968
10	Non-AD	Non-AD	0.040	0.960	0.000	-1.675	-0.275	-11.086	-17.454	11.694
11	Non-AD	Non-AD	0.039	0.961	0.000	-1.694	-0.292	-11.044	-17.449	11.973
12	Non-AD	**Mild to Moderate AD**	0.778	0.180	0.042	0.455	0.646	-13.249	-10.322	-7.412
13	Non-AD	**Mild to Moderate AD**	0.710	0.265	0.026	0.268	0.695	-13.241	-11.268	-6.611
14	Non-AD	Non-AD	0.053	0.947	0.000	-1.474	-0.098	-11.501	-17.249	8.779
15	Non-AD	Non-AD	0.048	0.952	0.000	-1.502	-0.473	-11.195	-17.159	10.731
16	Non-AD	Non-AD	0.282	0.718	0.000	-1.210	-0.657	-10.962	-12.830	8.515
17	Non-AD	Non-AD	0.037	0.963	0.000	-1.603	-0.324	-11.208	-17.733	11.133
18	Non-AD	Non-AD	0.100	0.900	0.000	-1.094	0.153	-12.126	-16.529	4.156
19	Mild to Moderate AD	**Non-AD**	0.041	0.959	0.000	-1.447	-0.357	-11.434	-17.714	9.624
20	Mild to Moderate AD	Mild to Moderate AD	0.877	0.004	0.119	0.567	1.280	-12.425	-1.581	-8.431
21	Mild to Moderate AD	**Non-AD**	0.042	0.958	0.000	-1.619	-0.229	-11.211	-17.464	10.871
22	Mild to Moderate AD	Mild to Moderate AD	0.840	0.000	0.160	0.506	1.482	-11.634	4.680	-8.316
23	Mild to Moderate AD	Mild to Moderate AD	0.589	0.000	0.411	2.640	1.526	-10.237	20.134	-9.514
24	Mild to Moderate AD	Mild to Moderate AD	0.613	0.000	0.387	1.435	0.430	-11.342	8.218	-10.426
25	Mild to Moderate AD	Mild to Moderate AD	0.916	0.001	0.083	0.154	1.330	-11.640	2.181	-6.842
26	Mild to Moderate AD	**Non-AD**	0.279	0.720	0.001	-0.464	0.454	-12.858	-14.756	-1.788
27	Mild to Moderate AD	Mild to Moderate AD	0.994	0.000	0.006	-0.295	-0.784	-9.573	6.659	0.740
28	Mild to Moderate AD	Mild to Moderate AD	0.687	0.000	0.313	1.614	1.070	-12.288	2.962	-10.715
29	Severe AD	Severe AD	0.000	0.000	1.000	4.143	-0.962	22.885	266.999	-9.108
30	Severe AD	Severe AD	0.000	0.000	1.000	3.686	-2.004	37.574	378.248	-10.414
31	Severe AD	Severe AD	0.269	0.000	0.731	1.438	-0.209	-7.928	32.561	-9.929
32	Severe AD	**Mild to Moderate AD**	0.838	0.002	0.160	0.741	1.366	-12.327	0.252	-9.018
33	Severe AD	Severe AD	0.092	0.000	0.908	1.992	3.249	-2.052	84.273	-6.637
34	Severe AD	Severe AD	0.045	0.000	0.955	2.136	-0.122	-5.161	55.877	-11.281
35	Severe AD	Severe AD	0.000	0.000	1.000	3.361	-1.785	28.913	312.914	-10.791
36	Severe AD	Severe AD	0.000	0.000	1.000	3.032	-2.124	31.882	334.641	-10.402

Table S15. Factor scores for clones 12III1 and 12CIII3. Probability to belonging to Non-AD, Mild-to-Moderate AD and Severe AD groups, and the squared Mahalanobis distances to the centroid of the group of tested 36 sera. Incorrectly discriminated sera are in bold.

Sera	Prior	Posterior	Pr(Mild to moderate AD)	Pr(Severe AD)	Pr(Non-AD)	F1	F2	D^2(Mild to moderate AD)	D^2(Severe AD)	D^2(Non-AD)
1	Non-AD	Non-AD	0.014	0.986	0.000	-2.352	-0.647	-9.356	-17.836	26.620
2	Non-AD	Non-AD	0.011	0.989	0.000	-2.149	-0.659	-9.676	-18.621	24.577
3	Non-AD	Non-AD	0.013	0.987	0.000	-2.302	-0.613	-9.491	-18.169	26.013
4	Non-AD	Non-AD	0.016	0.984	0.000	-2.273	-0.445	-9.761	-18.049	25.330
5	Non-AD	Non-AD	0.020	0.980	0.000	-2.164	-0.177	-10.235	-18.058	23.821
6	Non-AD	Non-AD	0.011	0.989	0.000	-2.233	-0.638	-9.571	-18.538	25.373
7	Non-AD	Non-AD	0.011	0.989	0.000	-2.260	-0.648	-9.513	-18.438	25.671
8	Non-AD	Non-AD	0.012	0.988	0.000	-2.148	-0.740	-9.559	-18.444	24.799
9	Non-AD	Non-AD	0.011	0.989	0.000	-2.247	-0.707	-9.449	-18.476	25.701
10	Non-AD	Non-AD	0.012	0.988	0.000	-2.278	-0.664	-9.459	-18.358	25.904
11	Non-AD	Non-AD	0.014	0.986	0.000	-2.342	-0.654	-9.363	-17.934	26.535
12	Non-AD	**Mild to Moderate AD**	0.607	0.393	0.000	-1.445	1.509	-11.486	-10.615	20.027
13	Non-AD	Non-AD	0.445	0.555	0.000	-1.279	1.228	-11.708	-12.149	17.430
14	Non-AD	Non-AD	0.025	0.975	0.000	-1.907	-0.591	-10.121	-17.431	22.012
15	Non-AD	**Mild to Moderate AD**	0.694	0.306	0.000	-1.608	-0.911	-10.023	-8.385	20.100
16	Non-AD	Non-AD	0.013	0.987	0.000	-2.061	-0.401	-10.144	-18.824	23.120
17	Non-AD	Non-AD	0.011	0.989	0.000	-2.214	-0.637	-9.603	-18.592	25.182
18	Non-AD	Non-AD	0.026	0.974	0.000	-1.807	-0.148	-10.755	-18.040	20.304
19	Mild to Moderate AD	**Non-AD**	0.015	0.985	0.000	-2.269	-0.447	-9.764	-18.085	25.296
20	Mild to Moderate AD	Mild to Moderate AD	0.815	0.185	0.000	-1.293	1.755	-11.438	-8.477	19.955
21	Mild to Moderate AD	**Non-AD**	0.013	0.987	0.000	-2.289	-0.581	-9.556	-18.211	25.803
22	Mild to Moderate AD	Mild to Moderate AD	0.856	0.144	0.000	-1.370	1.798	-11.358	-7.790	20.847
23	Mild to Moderate AD	Mild to Moderate AD	1.000	0.000	0.000	-0.580	3.711	-8.478	19.448	31.414
24	Mild to Moderate AD	Mild to Moderate AD	0.990	0.000	0.010	1.324	0.980	-10.480	175.680	-1.250
25	Mild to Moderate AD	Mild to Moderate AD	0.819	0.181	0.000	-1.044	1.183	-11.845	-8.825	15.289
26	Mild to Moderate AD	Mild to Moderate AD	0.853	0.000	0.147	2.232	-1.593	-6.794	498.784	-3.280
27	Mild to Moderate AD	Mild to Moderate AD	1.000	0.000	0.000	-0.758	-0.162	-11.539	15.640	11.085
28	Mild to Moderate AD	Mild to Moderate AD	0.999	0.000	0.001	0.871	1.637	-10.777	100.072	4.036
29	Severe AD	Severe AD	0.000	0.000	1.000	7.393	0.131	15.142	1943.691	-10.025
30	Severe AD	Severe AD	0.001	0.000	0.999	5.016	0.603	1.332	1013.966	-12.221
31	Severe AD	Severe AD	0.000	0.000	1.000	6.190	-1.917	9.269	1764.229	-8.268
32	Severe AD	Severe AD	0.099	0.000	0.901	3.173	-0.494	-5.727	617.911	-10.138
33	Severe AD	Severe AD	0.000	0.000	1.000	5.755	0.065	5.063	1319.896	-13.038
34	Severe AD	Severe AD	0.001	0.000	0.999	5.003	-0.469	1.429	1132.590	-13.176
35	Severe AD	Severe AD	0.001	0.000	0.999	4.981	0.338	1.112	1032.294	-12.802
36	Severe AD	Severe AD	0.001	0.000	0.999	4.734	0.004	-0.012	993.564	-13.083

Table S16. Factor scores for clones 12III15 and 12IV14. Probability to belonging to Non-AD, Mild-to-Moderate AD and Severe AD groups, and the squared Mahalanobis distances to the centroid of the group of tested 36 sera. Incorrectly discriminated sera are in bold.

Sera	Prior	Posterior	Pr(Mild to moderate AD)	Pr(Severe AD)	Pr(Non-AD)	F1	F2	D^2(Mild to moderate AD)	D^2(Severe AD)	D^2(Non-AD)
1	Non-AD	Non-AD	0.023	0.976	0.001	-1.321	0.101	-10.967	-18.447	-3.739
2	Non-AD	Non-AD	0.020	0.980	0.001	-1.308	0.059	-11.063	-18.883	-3.737
3	Non-AD	Non-AD	0.015	0.985	0.000	-1.474	-0.092	-10.785	-19.170	-2.972
4	Non-AD	Non-AD	0.198	0.799	0.003	-1.237	-0.596	-11.510	-14.301	-3.105
5	Non-AD	Non-AD	0.038	0.961	0.001	-0.917	-0.260	-12.065	-18.511	-4.660
6	Non-AD	Non-AD	0.014	0.985	0.000	-1.463	-0.060	-10.776	-19.236	-3.052
7	Non-AD	Non-AD	0.015	0.985	0.000	-1.473	-0.085	-10.778	-19.184	-2.985
8	Non-AD	Non-AD	0.015	0.985	0.000	-1.486	-0.080	-10.734	-19.111	-2.945
9	Non-AD	Non-AD	0.014	0.986	0.000	-1.433	-0.034	-10.829	-19.322	-3.191
10	Non-AD	Non-AD	0.014	0.985	0.000	-1.460	-0.065	-10.792	-19.257	-3.058
11	Non-AD	Non-AD	0.014	0.985	0.000	-1.466	-0.069	-10.780	-19.229	-3.033
12	Non-AD	Non-AD	0.350	0.637	0.013	-0.523	-0.369	-12.312	-13.514	-5.724
13	Non-AD	Non-AD	0.250	0.746	0.005	-1.026	-0.653	-11.738	-13.926	-3.755
14	Non-AD	Non-AD	0.022	0.978	0.001	-1.296	0.076	-11.071	-18.686	-3.794
15	Non-AD	Non-AD	0.019	0.980	0.000	-1.291	-0.267	-11.405	-19.271	-3.400
16	Non-AD	Non-AD	0.345	0.643	0.012	-0.640	-0.074	-12.398	-13.641	-5.708
17	Non-AD	Non-AD	0.017	0.983	0.000	-1.279	0.000	-11.213	-19.340	-3.769
18	Non-AD	Non-AD	0.014	0.986	0.000	-1.314	-0.085	-11.216	-19.728	-3.551
19	Mild to Moderate AD	**Non-AD**	0.019	0.980	0.000	-1.340	-0.259	-11.288	-19.126	-3.234
20	Mild to Moderate AD	Mild to Moderate AD	0.827	0.000	0.173	0.714	1.449	-11.879	157.147	-8.751
21	Mild to Moderate AD	**Non-AD**	0.021	0.979	0.001	-1.313	0.077	-11.024	-18.712	-3.738
22	Mild to Moderate AD	Mild to Moderate AD	0.906	0.000	0.094	-0.026	0.940	-12.252	55.689	-7.713
23	Mild to Moderate AD	Mild to Moderate AD	0.859	0.000	0.141	0.025	-1.180	-9.706	11.018	-6.091
24	Mild to Moderate AD	Mild to Moderate AD	0.626	0.000	0.374	1.314	0.318	-10.805	101.846	-9.775
25	Mild to Moderate AD	**Non-AD**	0.015	0.984	0.000	-1.308	-0.190	-11.320	-19.648	-3.440
26	Mild to Moderate AD	Mild to Moderate AD	0.661	0.000	0.339	1.449	2.458	-9.454	386.697	-8.118
27	Mild to Moderate AD	Mild to Moderate AD	0.950	0.009	0.041	-0.511	-1.019	-11.101	-1.827	-4.822
28	Mild to Moderate AD	Mild to Moderate AD	0.622	0.000	0.378	1.754	1.237	-10.747	232.817	-9.747
29	Severe AD	Severe AD	0.000	0.000	1.000	3.902	-1.191	17.394	308.967	-9.843
30	Severe AD	Severe AD	0.000	0.000	1.000	3.513	-2.174	23.706	262.993	-9.114
31	Severe AD	**Mild to Moderate AD**	0.696	0.000	0.304	0.250	-1.444	-7.879	26.378	-6.220
32	Severe AD	**Mild to Moderate AD**	0.602	0.000	0.398	1.991	2.691	-8.509	504.273	-7.681
33	Severe AD	**Mild to Moderate AD**	0.818	0.000	0.182	0.426	1.649	-11.234	157.926	-8.231
34	Severe AD	Severe AD	0.052	0.000	0.948	3.119	0.924	-4.009	368.393	-9.817
35	Severe AD	Severe AD	0.000	0.000	1.000	4.492	-0.588	18.415	426.363	-9.279
36	Severe AD	Severe AD	0.000	0.000	1.000	3.958	-1.148	17.604	317.889	-9.819

Table S17. Factor scores for clones 12III15 and 12CIII3. Probability to belonging to Non-AD, Mild-to-Moderate AD and Severe AD groups, and the squared Mahalanobis distances to the centroid of the group of tested 36 sera. Incorrectly discriminated sera are in bold.

Sera	Prior	Posterior	Pr(Mild to moderate AD)	Pr(Severe AD)	Pr(Non-AD)	F1	F2	D^2(Mild to moderate AD)	D^2(Severe AD)	D^2(Non-AD)
1	Non-AD	Non-AD	0.015	0.985	0.000	-2.061	-0.191	-11.761	-20.106	49.945
2	Non-AD	Non-AD	0.011	0.989	0.000	-1.889	-0.296	-11.726	-20.810	47.346
3	Non-AD	Non-AD	0.016	0.984	0.000	-2.087	-0.414	-11.399	-19.656	51.142
4	Non-AD	Non-AD	0.016	0.984	0.000	-2.097	-0.315	-11.558	-19.795	50.976
5	Non-AD	Non-AD	0.031	0.969	0.000	-1.986	0.091	-12.097	-19.011	47.758
6	Non-AD	Non-AD	0.012	0.988	0.000	-2.016	-0.433	-11.410	-20.185	49.981
7	Non-AD	Non-AD	0.013	0.987	0.000	-2.039	-0.439	-11.385	-19.997	50.399
8	Non-AD	Non-AD	0.012	0.988	0.000	-1.919	-0.524	-11.285	-20.073	48.627
9	Non-AD	Non-AD	0.011	0.989	0.000	-1.994	-0.404	-11.477	-20.418	49.503
10	Non-AD	Non-AD	0.013	0.987	0.000	-2.044	-0.414	-11.427	-20.055	50.400
11	Non-AD	Non-AD	0.017	0.983	0.000	-2.102	-0.387	-11.436	-19.581	51.312
12	Non-AD	Non-AD	0.242	0.758	0.000	-1.860	0.418	-12.307	-14.588	44.683
13	Non-AD	Non-AD	0.014	0.986	0.000	-1.784	-0.287	-11.798	-20.325	45.543
14	Non-AD	Non-AD	0.020	0.980	0.000	-1.708	-0.387	-11.658	-19.463	44.613
15	Non-AD	**Mild to Moderate AD**	0.551	0.449	0.000	-1.381	-0.697	-11.021	-10.612	40.336
16	Non-AD	Non-AD	0.190	0.810	0.000	-1.739	0.315	-12.390	-15.288	42.985
17	Non-AD	Non-AD	0.011	0.989	0.000	-1.948	-0.248	-11.762	-20.784	48.190
18	Non-AD	Non-AD	0.013	0.987	0.000	-1.790	-0.411	-11.575	-20.190	46.066
19	Mild to Moderate AD	**Non-AD**	0.016	0.984	0.000	-2.093	-0.317	-11.557	-19.834	50.918
20	Mild to Moderate AD	Mild to Moderate AD	1.000	0.000	0.000	-1.350	2.115	-9.626	63.257	33.193
21	Mild to Moderate AD	**Non-AD**	0.013	0.987	0.000	-2.031	-0.209	-11.759	-20.372	49.486
22	Mild to Moderate AD	Mild to Moderate AD	1.000	0.000	0.000	-1.661	1.301	-11.609	13.168	39.311
23	Mild to Moderate AD	Mild to Moderate AD	0.905	0.095	0.000	-1.802	0.719	-12.249	-7.752	42.914
24	Mild to Moderate AD	Mild to Moderate AD	1.000	0.000	0.000	1.134	0.891	-11.701	282.193	5.338
25	Mild to Moderate AD	**Non-AD**	0.047	0.953	0.000	-1.612	-0.549	-11.353	-17.355	43.567
26	Mild to Moderate AD	Mild to Moderate AD	0.597	0.000	0.403	2.945	0.673	-7.944	707.673	-7.158
27	Mild to Moderate AD	Mild to Moderate AD	1.000	0.000	0.000	-0.786	-0.443	-11.585	17.235	30.545
28	Mild to Moderate AD	Mild to Moderate AD	1.000	0.000	0.000	0.730	1.942	-10.777	262.666	8.253
29	Severe AD	Severe AD	0.000	0.000	1.000	6.977	-0.279	13.055	2347.975	-8.247
30	Severe AD	Severe AD	0.005	0.000	0.995	4.680	0.345	-1.290	1295.506	-12.059
31	Severe AD	Severe AD	0.000	0.000	1.000	5.694	-3.672	34.378	1803.381	-8.470
32	Severe AD	Severe AD	0.188	0.000	0.812	3.516	1.012	-6.639	898.118	-9.559
33	Severe AD	Severe AD	0.000	0.000	1.000	4.968	-1.977	12.698	1409.689	-10.945
34	Severe AD	Severe AD	0.002	0.000	0.998	5.170	0.666	0.041	1508.041	-12.033
35	Severe AD	Severe AD	0.007	0.000	0.993	5.121	1.714	-1.166	1541.278	-11.088
36	Severe AD	Severe AD	0.007	0.000	0.993	4.844	1.094	-2.025	1390.935	-11.815

Table S18. Factor scores for clones 12CIII3 and 12IV14. Probability to belonging to Non-AD, Mild-to-Moderate AD and Severe AD groups, and the squared Mahalanobis distances to the centroid of the group of tested 36 sera. Incorrectly discriminated sera are in bold.

Sera	Prior	Posterior	Pr(Mild to moderate AD)	Pr(Severe AD)	Pr(Non-AD)	F1	F2	D^2(Mild to moderate AD)	D^2(Severe AD)	D^2(Non-AD)
1	Non-AD	Non-AD	0.025	0.975	0.000	-2.070	-0.205	-11.828	-19.119	22.632
2	Non-AD	Non-AD	0.018	0.982	0.000	-1.881	-0.246	-12.046	-20.007	20.788
3	Non-AD	Non-AD	0.022	0.978	0.000	-2.033	-0.197	-11.914	-19.493	22.293
4	Non-AD	Non-AD	0.054	0.946	0.000	-1.925	0.323	-12.809	-18.553	22.330
5	Non-AD	Non-AD	0.054	0.946	0.000	-1.925	0.323	-12.809	-18.553	22.330
6	Non-AD	Non-AD	0.019	0.981	0.000	-1.969	-0.241	-11.913	-19.796	21.613
7	Non-AD	Non-AD	0.019	0.981	0.000	-1.986	-0.223	-11.931	-19.767	21.800
8	Non-AD	Non-AD	0.020	0.980	0.000	-1.861	-0.292	-11.961	-19.788	20.524
9	Non-AD	Non-AD	0.019	0.981	0.000	-1.956	-0.243	-11.931	-19.851	21.484
10	Non-AD	Non-AD	0.020	0.980	0.000	-1.997	-0.222	-11.914	-19.698	21.908
11	Non-AD	Non-AD	0.024	0.976	0.000	-2.056	-0.198	-11.872	-19.283	22.505
12	Non-AD	Non-AD	0.264	0.736	0.000	-1.794	0.659	-12.809	-14.854	21.856
13	Non-AD	Non-AD	0.122	0.878	0.000	-1.600	0.390	-13.099	-17.049	19.480
14	Non-AD	Non-AD	0.036	0.964	0.000	-1.698	-0.331	-12.113	-18.673	18.979
15	Non-AD	**Mild to Moderate AD**	0.790	0.210	0.000	-1.266	-0.259	-12.804	-10.154	15.356
16	Non-AD	Non-AD	0.055	0.945	0.000	-1.739	0.319	-12.992	-18.698	20.594
17	Non-AD	Non-AD	0.018	0.982	0.000	-1.929	-0.159	-12.178	-20.187	21.389
18	Non-AD	Non-AD	0.025	0.975	0.000	-1.739	-0.206	-12.359	-19.698	19.567
19	Mild to Moderate AD	**Non-AD**	0.025	0.975	0.000	-2.004	0.022	-12.408	-19.702	22.450
20	Mild to Moderate AD	**Non-AD**	0.043	0.957	0.000	-1.847	0.288	-12.879	-19.100	21.524
21	Mild to Moderate AD	**Non-AD**	0.022	0.978	0.000	-2.033	-0.197	-11.914	-19.493	22.293
22	Mild to Moderate AD	**Non-AD**	0.032	0.968	0.000	-1.978	0.145	-12.613	-19.447	22.447
23	Mild to Moderate AD	Mild to Moderate AD	1.000	0.000	0.000	-1.555	1.599	-10.017	9.861	22.071
24	Mild to Moderate AD	Mild to Moderate AD	0.997	0.000	0.003	0.994	0.359	-11.031	189.232	0.488
25	Mild to Moderate AD	**Non-AD**	0.102	0.898	0.000	-1.525	-0.216	-12.615	-16.957	17.650
26	Mild to Moderate AD	Mild to Moderate AD	0.598	0.000	0.402	2.287	-1.722	-7.250	497.405	-6.453
27	Mild to Moderate AD	Mild to Moderate AD	1.000	0.000	0.000	-0.503	0.588	-12.791	27.961	10.756
28	Mild to Moderate AD	Mild to Moderate AD	1.000	0.000	0.000	0.293	0.323	-12.422	98.572	4.675
29	Severe AD	Severe AD	0.000	0.000	1.000	7.279	0.765	29.945	2143.838	-8.743
30	Severe AD	Severe AD	0.000	0.000	1.000	5.187	2.124	26.926	1258.623	-10.026
31	Severe AD	Severe AD	0.000	0.000	1.000	6.287	-1.494	8.626	1806.790	-9.468
32	Severe AD	Severe AD	0.321	0.000	0.679	2.777	-1.686	-6.429	612.354	-7.930
33	Severe AD	Severe AD	0.001	0.000	0.999	4.684	-2.981	4.891	1297.552	-8.985
34	Severe AD	Severe AD	0.001	0.000	0.999	4.889	-0.394	2.587	1189.133	-11.359
35	Severe AD	Severe AD	0.000	0.000	1.000	5.144	1.721	21.323	1239.839	-10.483
36	Severe AD	Severe AD	0.000	0.000	1.000	5.048	1.764	21.036	1205.776	-10.381

Table S19. Factor scores for clones 12CIII1/12CIII3 and 12III1. Probability to belonging to Non-AD, Mild-to-Moderate AD and Severe AD groups, and the squared Mahalanobis distances to the centroid of the group of tested 36 sera. Incorrectly discriminated sera are in bold.

Sera	Prior	Posterior	Pr(Mild to moderate AD)	Pr(Severe AD)	Pr(Non-AD)	F1	F2	D²(Mild to moderate AD)	D²(Severe AD)	D²(Non-AD)
1	Non-AD	Non-AD	0.002	0.998	0.000	-2.551	-0.567	-16.428	-28.661	43.173
2	Non-AD	Non-AD	0.002	0.998	0.000	-2.318	-0.533	-16.832	-29.549	39.393
3	Non-AD	Non-AD	0.002	0.998	0.000	-2.524	-0.605	-16.469	-28.799	42.806
4	Non-AD	Non-AD	0.002	0.998	0.000	-2.455	-0.411	-16.795	-28.961	38.894
5	Non-AD	Non-AD	0.119	0.881	0.000	-2.220	0.035	-17.513	-21.516	30.692
6	Non-AD	Non-AD	0.002	0.998	0.000	-2.464	-0.649	-16.527	-29.081	42.455
7	Non-AD	Non-AD	0.002	0.998	0.000	-2.482	-0.633	-16.505	-29.061	42.675
8	Non-AD	Non-AD	0.002	0.998	0.000	-2.371	-0.708	-16.585	-29.049	42.361
9	Non-AD	Non-AD	0.002	0.998	0.000	-2.459	-0.651	-16.498	-29.150	42.955
10	Non-AD	Non-AD	0.002	0.998	0.000	-2.475	-0.583	-16.541	-29.262	42.261
11	Non-AD	Non-AD	0.002	0.998	0.000	-2.551	-0.596	-16.407	-28.622	43.479
12	Non-AD	Non-AD	0.021	0.979	0.000	-2.009	-0.141	-13.841	-21.564	24.513
13	Non-AD	Non-AD	0.014	0.986	0.000	-1.827	-0.303	-14.620	-23.079	23.187
14	Non-AD	Non-AD	0.039	0.961	0.000	-1.979	-0.299	-17.471	-23.873	32.115
15	Non-AD	Non-AD	0.244	0.756	0.000	-1.834	-0.892	-17.089	-19.355	37.332
16	Non-AD	Non-AD	0.002	0.998	0.000	-2.306	-0.553	-16.916	-29.794	37.494
17	Non-AD	Non-AD	0.003	0.997	0.000	-2.335	-0.399	-16.876	-28.770	38.403
18	Non-AD	Non-AD	0.004	0.996	0.000	-2.175	-0.704	-16.716	-27.894	35.204
19	Mild to Moderate AD	Mild to Moderate AD	0.998	0.002	0.000	-2.127	0.327	-17.235	-5.250	29.467
20	Mild to Moderate AD	Mild to Moderate AD	1.000	0.000	0.000	-1.176	1.551	-18.438	81.773	12.403
21	Mild to Moderate AD	Mild to Moderate AD	0.998	0.002	0.000	-2.122	0.302	-16.990	-4.569	30.420
22	Mild to Moderate AD	Mild to Moderate AD	1.000	0.000	0.000	-0.260	3.849	-15.948	558.579	19.875
23	Mild to Moderate AD	Mild to Moderate AD	1.000	0.000	0.000	0.238	4.304	-15.847	615.110	39.635
24	Mild to Moderate AD	Mild to Moderate AD	0.986	0.000	0.014	1.657	1.214	-17.965	407.017	-9.448
25	Mild to Moderate AD	**Non-AD**	0.080	0.920	0.000	-1.657	-0.510	-14.089	-18.975	22.424
26	Mild to Moderate AD	Mild to Moderate AD	0.998	0.000	0.002	2.289	-1.168	-14.259	556.322	-1.494
27	Mild to Moderate AD	Mild to Moderate AD	1.000	0.000	0.000	-0.431	0.739	-18.845	110.226	6.556
28	Mild to Moderate AD	Mild to Moderate AD	1.000	0.000	0.000	1.310	1.932	-18.259	398.959	-3.012
29	Severe AD	Severe AD	0.000	0.000	1.000	7.434	-0.761	8.066	2291.368	-17.617
30	Severe AD	Severe AD	0.002	0.000	0.998	5.383	0.584	-6.147	1438.309	-18.312
31	Severe AD	Severe AD	0.000	0.000	1.000	6.796	-0.626	3.867	2208.079	-16.497
32	Severe AD	Severe AD	0.038	0.000	0.962	3.930	1.007	-11.129	1072.751	-17.590
33	Severe AD	Severe AD	0.000	0.000	1.000	5.561	-1.130	-1.138	1479.549	-20.658
34	Severe AD	Severe AD	0.001	0.000	0.999	5.167	-0.560	-6.051	1377.480	-21.242
35	Severe AD	Severe AD	0.001	0.000	0.999	4.583	-1.324	-3.508	1112.058	-18.665
36	Severe AD	Severe AD	0.001	0.000	0.999	4.760	-0.538	-7.200	1193.686	-21.066

Table S20. Factor scores for clones 12CIII1/12CIII3 and 12III15. Probability to belonging to Non-AD, Mild-to-Moderate AD and Severe AD groups, and the squared Mahalanobis distances to the centroid of the group of tested 36 sera. Incorrectly discriminated sera are in bold.

Sera	Prior	Posterior	Pr(Mild to moderate AD)	Pr(Severe AD)	Pr(Non-AD)	F1	F2	D^2(Mild to moderate AD)	D^2(Severe AD)	D^2(Non-AD)
1	Non-AD	Non-AD	0.001	0.999	0.000	-2.514	-0.538	-16.876	-30.698	76.294
2	Non-AD	Non-AD	0.001	0.999	0.000	-2.293	-0.526	-17.385	-31.917	71.517
3	Non-AD	Non-AD	0.001	0.999	0.000	-2.532	-0.619	-16.955	-30.650	78.499
4	Non-AD	Non-AD	0.001	0.999	0.000	-2.463	-0.423	-17.253	-30.587	75.047
5	Non-AD	Non-AD	0.030	0.970	0.000	-2.180	0.068	-17.864	-24.832	63.702
6	Non-AD	Non-AD	0.001	0.999	0.000	-2.473	-0.664	-17.003	-31.066	77.480
7	Non-AD	Non-AD	0.001	0.999	0.000	-2.489	-0.650	-16.999	-30.962	77.806
8	Non-AD	Non-AD	0.001	0.999	0.000	-2.380	-0.734	-17.082	-30.993	76.221
9	Non-AD	Non-AD	0.001	0.999	0.000	-2.452	-0.657	-17.031	-31.251	76.755
10	Non-AD	Non-AD	0.001	0.999	0.000	-2.472	-0.596	-17.083	-31.147	76.909
11	Non-AD	Non-AD	0.001	0.999	0.000	-2.546	-0.603	-16.933	-30.555	78.606
12	Non-AD	Non-AD	0.012	0.988	0.000	-2.216	-0.116	-16.764	-25.644	63.667
13	Non-AD	Non-AD	0.002	0.998	0.000	-2.110	-0.397	-17.803	-30.412	66.466
14	Non-AD	Non-AD	0.015	0.985	0.000	-1.965	-0.326	-18.055	-26.453	63.337
15	Non-AD	Non-AD	0.109	0.891	0.000	-1.851	-0.937	-17.351	-21.552	66.158
16	Non-AD	Non-AD	0.036	0.964	0.000	-2.226	-0.436	-16.471	-23.056	66.257
17	Non-AD	Non-AD	0.001	0.999	0.000	-2.298	-0.390	-17.514	-31.428	70.559
18	Non-AD	Non-AD	0.001	0.999	0.000	-2.267	-0.737	-17.212	-30.681	72.903
19	Mild to Moderate AD	Mild to Moderate AD	1.000	0.000	0.000	-2.089	0.277	-18.326	-1.955	63.244
20	Mild to Moderate AD	Mild to Moderate AD	1.000	0.000	0.000	-1.042	1.816	-14.229	91.335	29.673
21	Mild to Moderate AD	Mild to Moderate AD	0.998	0.002	0.000	-2.045	0.282	-18.425	-6.282	61.603
22	Mild to Moderate AD	Mild to Moderate AD	1.000	0.000	0.000	-0.125	3.838	-17.579	544.434	32.358
23	Mild to Moderate AD	Mild to Moderate AD	1.000	0.000	0.000	-0.022	4.062	-15.923	720.398	37.129
24	Mild to Moderate AD	Mild to Moderate AD	1.000	0.000	0.000	1.713	1.280	-18.457	392.054	-2.423
25	Mild to Moderate AD	**Non-AD**	0.006	0.994	0.000	-1.987	-0.644	-17.657	-28.013	66.539
26	Mild to Moderate AD	Mild to Moderate AD	0.972	0.000	0.028	2.611	-0.911	-14.889	697.262	-7.766
27	Mild to Moderate AD	Mild to Moderate AD	1.000	0.000	0.000	-0.432	0.625	-18.325	104.897	30.632
28	Mild to Moderate AD	Mild to Moderate AD	1.000	0.000	0.000	1.468	2.142	-17.818	391.413	0.018
29	Severe AD	Severe AD	0.000	0.000	1.000	7.345	-0.814	13.758	2417.698	-15.666
30	Severe AD	Severe AD	0.000	0.000	1.000	5.389	0.576	-3.392	1452.090	-18.968
31	Severe AD	Severe AD	0.000	0.000	1.000	6.509	-1.259	59.194	2158.659	-16.671
32	Severe AD	Severe AD	0.069	0.000	0.931	4.255	1.168	-11.618	1047.229	-16.823
33	Severe AD	Severe AD	0.000	0.000	1.000	5.176	-1.492	15.584	1484.141	-17.536
34	Severe AD	Severe AD	0.001	0.000	0.999	5.340	-0.398	-5.530	1526.946	-20.251
35	Severe AD	Severe AD	0.009	0.000	0.991	4.741	-0.953	-7.957	1538.967	-17.324
36	Severe AD	Severe AD	0.004	0.000	0.996	4.923	-0.313	-8.654	1394.193	-19.895

Table S21. Factor scores for clones 12CIII1/12CIII3 and 12IV14. Probability to belonging to Non-AD, Mild-to-Moderate AD and Severe AD groups, and the squared Mahalanobis distances to the centroid of the group of tested 36 sera. Incorrectly discriminated sera are in bold.

Sera	Prior	Posterior	Pr(Mild to moderate AD)	Pr(Severe AD)	Pr(Non-AD)	F1	F2	D^2(Mild to moderate AD)	D^2(Severe AD)	D^2(Non-AD)
1	Non-AD	Non-AD	0.002	0.998	0.000	-2.515	-0.542	-17.667	-30.193	31.171
2	Non-AD	Non-AD	0.002	0.998	0.000	-2.283	-0.493	-18.088	-30.922	28.056
3	Non-AD	Non-AD	0.002	0.998	0.000	-2.494	-0.586	-17.644	-30.421	31.151
4	Non-AD	Non-AD	0.002	0.998	0.000	-2.360	-0.508	-16.908	-29.380	28.970
5	Non-AD	Non-AD	0.042	0.958	0.000	-2.145	-0.038	-18.129	-24.399	24.436
6	Non-AD	Non-AD	0.002	0.998	0.000	-2.439	-0.622	-17.698	-30.688	30.680
7	Non-AD	Non-AD	0.002	0.998	0.000	-2.451	-0.609	-17.691	-30.684	30.751
8	Non-AD	Non-AD	0.002	0.998	0.000	-2.339	-0.668	-17.801	-30.678	29.720
9	Non-AD	Non-AD	0.001	0.999	0.000	-2.423	-0.620	-17.726	-30.783	30.466
10	Non-AD	Non-AD	0.001	0.999	0.000	-2.439	-0.553	-17.778	-30.807	30.289
11	Non-AD	Non-AD	0.002	0.998	0.000	-2.513	-0.574	-17.628	-30.239	31.332
12	Non-AD	Non-AD	0.006	0.994	0.000	-2.181	-0.401	-15.724	-25.958	26.356
13	Non-AD	Non-AD	0.004	0.996	0.000	-2.001	-0.497	-16.956	-28.180	24.600
14	Non-AD	Non-AD	0.099	0.901	0.000	-1.954	-0.221	-18.746	-23.172	22.903
15	Non-AD	Non-AD	0.204	0.796	0.000	-1.776	-0.863	-18.151	-20.876	24.075
16	Non-AD	Non-AD	0.003	0.997	0.000	-2.227	-0.663	-16.642	-28.513	28.120
17	Non-AD	Non-AD	0.002	0.998	0.000	-2.282	-0.370	-18.215	-30.331	27.408
18	Non-AD	Non-AD	0.002	0.998	0.000	-2.230	-0.716	-17.851	-30.354	28.612
19	Mild to Moderate AD	Mild to Moderate AD	0.999	0.001	0.000	-2.036	0.365	-19.133	-5.361	21.909
20	Mild to Moderate AD	Mild to Moderate AD	1.000	0.000	0.000	-1.350	1.511	-20.044	111.210	14.469
21	Mild to Moderate AD	Mild to Moderate AD	1.000	0.000	0.000	-2.044	0.398	-18.939	-0.167	21.855
22	Mild to Moderate AD	Mild to Moderate AD	1.000	0.000	0.000	-0.331	4.041	-14.664	710.115	19.647
23	Mild to Moderate AD	Mild to Moderate AD	1.000	0.000	0.000	0.096	4.085	-16.860	679.570	22.431
24	Mild to Moderate AD	Mild to Moderate AD	0.995	0.000	0.005	1.620	1.185	-18.071	403.144	-7.663
25	Mild to Moderate AD	**Non-AD**	0.009	0.991	0.000	-1.930	-0.575	-18.432	-27.757	24.254
26	Mild to Moderate AD	Mild to Moderate AD	0.932	0.000	0.068	2.233	-0.762	-14.153	561.200	-8.919
27	Mild to Moderate AD	Mild to Moderate AD	1.000	0.000	0.000	-0.270	0.670	-18.810	94.235	4.607
28	Mild to Moderate AD	Mild to Moderate AD	1.000	0.000	0.000	1.193	1.934	-19.493	423.038	-3.011
29	Severe AD	Severe AD	0.000	0.000	1.000	7.519	-0.982	29.506	2295.057	-16.620
30	Severe AD	Severe AD	0.000	0.000	1.000	5.671	0.121	30.730	1431.964	-16.710
31	Severe AD	Severe AD	0.000	0.000	1.000	6.881	-0.191	2.027	2160.322	-17.277
32	Severe AD	Severe AD	0.027	0.000	0.973	3.820	1.535	-7.870	1150.340	-15.024
33	Severe AD	Severe AD	0.000	0.000	1.000	5.036	-0.501	2.799	1558.527	-16.099
34	Severe AD	Severe AD	0.000	0.000	1.000	5.174	-0.467	-3.936	1352.882	-19.422
35	Severe AD	Severe AD	0.000	0.000	1.000	4.741	-1.843	34.160	1233.501	-16.118
36	Severe AD	Severe AD	0.000	0.000	1.000	5.028	-0.981	28.338	1246.202	-18.333

Table S22. Factor scores obtained by using all clones simultaneously. Probability to belonging to Non-AD, Mild-to-Moderate AD and Severe AD groups, and the squared Mahalanobis distances to the centroid of the group of tested 36 sera.

Sera	Prior	Posterior	Pr(Mild to moderate AD)	Pr(Severe AD)	Pr(Non-AD)	F1	F2	D^2(Mild to moderate AD)	D^2(Severe AD)	D^2(Non-AD)
1	Non-AD	Non-AD	0.000	1.000	0.000	-2.551	-0.535	-33.607	-50.068	99.780
2	Non-AD	Non-AD	0.000	1.000	0.000	-2.329	-0.524	-34.276	-51.200	93.380
3	Non-AD	Non-AD	0.000	1.000	0.000	-2.549	-0.646	-33.921	-50.970	104.215
4	Non-AD	Non-AD	0.007	0.993	0.000	-2.442	-0.648	-32.118	-41.929	104.550
5	Non-AD	Non-AD	0.005	0.995	0.000	-2.187	-0.085	-33.041	-43.538	86.639
6	Non-AD	Non-AD	0.000	1.000	0.000	-2.492	-0.676	-34.030	-51.350	102.339
7	Non-AD	Non-AD	0.000	1.000	0.000	-2.508	-0.672	-33.985	-51.291	103.095
8	Non-AD	Non-AD	0.000	1.000	0.000	-2.402	-0.748	-34.121	-51.333	100.632
9	Non-AD	Non-AD	0.000	1.000	0.000	-2.478	-0.673	-33.949	-51.530	101.243
10	Non-AD	Non-AD	0.000	1.000	0.000	-2.497	-0.621	-34.015	-51.462	101.858
11	Non-AD	Non-AD	0.000	1.000	0.000	-2.570	-0.632	-33.816	-50.893	104.281
12	Non-AD	Non-AD	0.002	0.998	0.000	-2.063	-0.119	-29.558	-42.494	96.247
13	Non-AD	Non-AD	0.004	0.996	0.000	-1.950	-0.418	-31.826	-42.824	100.846
14	Non-AD	Non-AD	0.013	0.987	0.000	-2.010	-0.305	-35.022	-43.641	82.056
15	Non-AD	Non-AD	0.047	0.953	0.000	-1.861	-1.004	-34.282	-40.320	87.635
16	Non-AD	Non-AD	0.004	0.996	0.000	-2.230	-0.552	-29.652	-40.659	87.250
17	Non-AD	Non-AD	0.000	1.000	0.000	-2.336	-0.424	-34.174	-50.927	92.939
18	Non-AD	Non-AD	0.000	1.000	0.000	-2.239	-0.688	-34.278	-49.674	95.767
19	Mild to Moderate AD	Mild to Moderate AD	0.999	0.001	0.000	-2.137	0.139	-34.659	-21.019	86.290
20	Mild to Moderate AD	Mild to Moderate AD	1.000	0.000	0.000	-1.100	2.161	-31.160	226.181	38.644
21	Mild to Moderate AD	Mild to Moderate AD	0.999	0.001	0.000	-2.126	0.237	-34.563	-20.670	81.094
22	Mild to Moderate AD	Mild to Moderate AD	1.000	0.000	0.000	-0.280	3.998	-30.816	708.391	48.901
23	Mild to Moderate AD	Mild to Moderate AD	1.000	0.000	0.000	0.109	3.747	-31.948	710.275	112.470
24	Mild to Moderate AD	Mild to Moderate AD	1.000	0.000	0.000	1.704	1.323	-34.561	446.551	-6.628
25	Mild to Moderate AD	Mild to Moderate AD	0.503	0.497	0.000	-1.850	-0.436	-29.769	-29.742	96.263
26	Mild to Moderate AD	Mild to Moderate AD	0.999	0.000	0.001	2.382	-0.220	-30.494	976.313	-15.693
27	Mild to Moderate AD	Mild to Moderate AD	1.000	0.000	0.000	-0.420	0.238	-33.122	105.026	45.360
28	Mild to Moderate AD	Mild to Moderate AD	1.000	0.000	0.000	1.402	2.407	-34.546	561.723	-2.173
29	Severe AD	Severe AD	0.000	0.000	1.000	7.519	-1.126	15.698	2543.331	-30.589
30	Severe AD	Severe AD	0.000	0.000	1.000	5.582	-0.246	18.592	1645.677	-30.933
31	Severe AD	Severe AD	0.000	0.000	1.000	6.519	-1.387	52.165	2195.287	-30.294
32	Severe AD	Severe AD	0.007	0.000	0.993	3.975	1.922	-21.209	1554.462	-31.018
33	Severe AD	Severe AD	0.000	0.000	1.000	5.156	-0.416	56.544	1769.628	-30.235
34	Severe AD	Severe AD	0.001	0.000	0.999	5.290	-0.163	-20.070	1701.047	-34.630
35	Severe AD	Severe AD	0.000	0.000	1.000	4.912	-1.324	30.275	1672.099	-32.677
36	Severe AD	Severe AD	0.000	0.000	1.000	5.058	-0.886	20.423	1559.858	-34.597
